# Selective accumulation of pharmaceutical residues from 6 different soils by plants: a comparative study on onion, radish, and spinach

**DOI:** 10.1007/s11356-023-26102-5

**Published:** 2023-03-04

**Authors:** Sunil Paul M. Menacherry, Radka Kodešová, Helena Švecová, Aleš Klement, Miroslav Fér, Antonín Nikodem, Roman Grabic

**Affiliations:** 1 Department of Soil Science and Soil Protection, Faculty of Agrobiology, Food and Natural Resources, Czech University of Life Sciences Prague, Kamýcká 129, 16500 Prague 6, Czech Republic; 2 South Bohemian Research Center of Aquaculture and Biodiversity of Hydrocenoses, Faculty of Fisheries and Protection of Waters, University of South Bohemia in České Budějovice, Zátiší 728/II, 38925 Vodňany, Czech Republic

**Keywords:** Pharmaceuticals, Plants, Soils, Metabolism, Root uptake, Pharmaceutical accumulation, Translocation of pharmaceuticals in plant, Plant-dependent transformation of pharmaceuticals

## Abstract

The accumulation of six pharmaceuticals of different therapeutic uses has been thoroughly investigated and compared between onion, spinach, and radish plants grown in six soil types. While neutral molecules (e.g., carbamazepine (CAR) and some of its metabolites) were efficiently accumulated and easily translocated to the plant leaves (onion > radish > spinach), the same for ionic (both anionic and cationic) molecules seems to be minor to moderate. The maximum accumulation of CAR crosses 38,000 (onion), 42,000 (radish), and 7000 (spinach) ng g^−1^ (dry weight) respectively, in which the most majority of them happened within the plant leaves. Among the metabolites, the accumulation of carbamazepine 10,11-epoxide (EPC — a primary CAR metabolite) was approximately 19,000 (onion), 7000 (radish), and 6000 (spinach) ng g^−1^ (dry weight) respectively. This trend was considerably similar even when all these pharmaceuticals applied together. The accumulation of most other molecules (e.g., citalopram, clindamycin, clindamycin sulfoxide, fexofenadine, irbesartan, and sulfamethoxazole) was restricted to plant roots, except for certain cases (e.g., clindamycin and clindamycin sulfoxide in onion leaves). Our results clearly demonstrated the potential role of this accumulation process on the entrance of pharmaceuticals/metabolites into the food chain, which eventually becomes a threat to associated living biota.

**Supplementary Information:**

The online version contains supplementary material available at 10.1007/s11356-023-26102-5.

## Introduction

Pharmaceuticals, a group of molecules designed to induce specific biological activities on targeted organisms, are increasingly reported to exist in the precious environmental resources (Kümmerer 2009; Mezzelani et al. 2018; Papageorgiou et al. 2016; Yan et al. 2013). This eventually led to categorize many pharmaceuticals as contaminants of emerging concern (CECs) by various environmental agencies as these molecules might induce unprecedented impacts on living biota. The capability of many pharmaceuticals to resist microbial and most traditional wastewater treatment protocols up to certain extends is documented (Fatta-Kassinos et al. 2020; Goñi-Urriza et al. 2000; Watkinson et al. 2007; Zhang et al. 2009). The use of treated wastewater for irrigation and soil amendment purposes led to the spreading of these molecules from aquatic environment to the natural soils (Biel-Maeso et al. 2018; Iwane et al. 2001; Ma et al. 2018; Poustie et al. 2020; Wu et al. 2015; Xia et al. 2005). It is a known fact that the molecules, including pharmaceuticals, from soil can be taken up by plants via their transpiration stream (Brunetti et al. 2022; Dodgen et al. 2015; Kodešová et al. 2019a, b; Kumar and Gupta 2016). These molecules might be then transported across the plant cells, accumulated on the edible plant parts (led to their entrance into food chain), or even metabolized into structurally similar molecules with unknown toxicological effects (Ben Mordechay et al. 2022; Brunetti et al. 2021; Klampfl 2019; Kodešová et al. 2019a, b; Wu et al. 2010). This important environmental scenario is, however, very little explored by the scientific community, which necessitates extensive experimental efforts to fully understand their environmental implications.

A number of physical and chemical parameters, such as the ionic strength and molecular mass of the molecule, its hydrophobicity and solubility, lipophilicity and ionization behavior (e.g., dissociation constant-p*K*_a_), pH of the environment, and its susceptibility towards various processes including sorption and biodegradation, are known to influence the potential transport of a molecule from the external environment into the plant roots and other organelles (Collins et al. 2006, 2011; Kurade et al. 2021). Moreover, this process is also identified as plant and soil dependent (Brunetti et al. 2021; Klement et al. 2020; Kodešová et al. 2019b; Miller et al. 2016). It is, thus, expected that the pharmaceuticals with unique structural features might be variably taken up by different plants grown in different soil environments (Ben Mordechay et al. 2022). Moreover, significant differences in the transport and metabolism of individual pharmaceuticals within the plant compartment are also expected. While neutral molecules are known to diffuse through the plant cell membranes much easily, the same for charged species might be slowed down due to the repulsion (cationic)/binding (anionic) with the negatively charged cell walls (Ben Mordechay et al. 2022; García and Fernández-López 2022; Goldstein et al. 2014; Khalaf et al. 2022; Klement et al. 2020; Kodešová et al. 2019b; Malchi et al. 2014; Pérez et al. 2022; Rhodes et al. 2021). This behavior is clearly demonstrated in the case of a neutral molecule, carbamazepine, where their translocation from the roots to edible parts of plants (e.g., leaves and fruits) seems extremely efficient in lamb’s lettuce, spinach, arugula, radish, green pea, leafy greens, potatoes, carrots, bananas, tomatoes, avocados, citrus, and corn (Ben Mordechay et al. 2022; Klement et al. 2020; Kodešová et al. 2019b; Pérez et al. 2022). Another important factor which likely influence the fate of a specific molecule before (i.e., in soil) or after (i.e., in plant tissues) the uptake is the presence of other chemical species, such as micronutrients, heavy metals, simple organic molecules, and other pharmaceuticals (Beltrán et al. 2021; Khalaf et al. 2022; Kodešová et al. 2020; Liang et al. 2019; Papaioannou et al. 2019; Zheng and Guo 2021). On the other hand, either alone or as a mixture, many of these biomolecules might affect the plant health after being taken up from the environment via the transpiration stream, especially in the case of higher exposure levels. A recent study by Akenga et al. (2021) clearly revealed the toxic effects of antiretroviral medications, such as nevirapine, on lettuce upon higher exposure levels (≥ 100 μg L^−1^). Furthermore, this drug seems to move flawlessly from the plant root to the edible parts (e.g., leaves) with a translocation factor > 1. Another study by Alaoui and coworkers noticed the bioaccumulation of pharmaceuticals (e.g. carbamazepine and irbesartan) on an aquatic moss *Fontinalis antipyretica*, which eventually disturb the redox status of the plant, but no noticeable effect on its growth (Sossey Alaoui et al. 2021).

Despite the possible health effects likely occurred to the directly affected plants as well as any other living species consuming them as a part of their food chain, in-depth studies on the uptake of pharmaceuticals and their consequent translocation/accumulation into the edible parts, such as the leaves and fruits, are not much available. To explore the above-explained environmental scenarios, the uptakes of a number of pharmaceuticals by different plant species (onion, spinach, and radish) planted in a series of soil types were systematically investigated. The selected pharmaceuticals, which includes both neutral and ionic molecules, are carbamazepine (neutral), citalopram (cation), clindamycin (cation and neutral), sulfamethoxazole (neutral, anion), fexofenadine (cation, zwitter-ion, and anion), and irbesartan (cation, zwitter-ion or neutral, and anion). In addition, the molar fractions (i.e., the proportion of a specific molecule out of the entire pharmaceuticals and metabolites in percentage) and bio-accumulation factors (i.e., the ratios of each pharmaceutical and their metabolites between plants and soils) were also calculated and compared to each other with an aim to better understand the implications of this important environmental process.

## Experimental

### Chemicals

A total of six parent pharmaceuticals and some of their metabolites were purchased commercially and used as received. Isotopically labeled analogs of most of the parent compounds were also purchased from Toronto Research Chemicals (Canada) and used as internal standards. All other reagents, including acetonitrile (LC-MS grade: Merck, Darmstadt, Germany), 2-propanol (LiChrosolv Hypergrade: Merck, Darmstadt, Germany), and formic acid (LC-MS grade: Labicom, Olomouc, Czech Republic), used for this study were of the highest available purities. The ultrapure water prepared using an Aqua-MAX-Ultra System (Younglin, Kyounggi-do, South Korea) was used to prepare the analytical standards. A stock solution of individual pharmaceuticals was prepared in methanol at an initial concentration of 1 mg/mL. Analytical working solutions of these individual compounds as well as their mixtures were prepared by further diluting the stock solutions with methanol to a final concentration of 1 µg/mL. All the stock and working solutions were stored at −20 °C. More details of the selected drugs and their metabolites are available in Table 1.

**Table 1:** The details of parent and metabolites of selected pharmaceuticals (data obtained from Ref. Klement et al. 2018; Kodešová et al. 2019a, b), ^a^http://www.chemspider.com/, ^b^https://www.guidechem.com/, and ^c^https://hmdb.ca/)

Compound	Abbreviation	CAS	LogK_ow_	p*K*_a_	MW (g/mol)
Carbamazepine	CAR	298–46-4	2.25	pKa_1_ = 1.00 (basic), pKa_2_ = 13.90 (acidic)	236.27
Carbamazepine 10,11-epoxide	EPC	36507–30-9	0.95^a^	pKa_1_ = −3.70 (basic), pKa_2_ = 15.96 (acidic)	252.27
*rac trans*-10,11-dihydro-10,11-dihydroxy carbamazepine	TDC	58955–93-4	0.71	pKa = 12.70 (acidic)	270.29
10,11-Dihydrocarbamazepine	DHC	3564–73-6	2.46^a^	pKa = 14.07^b^	238.28
Oxcarbazepine	OXC	28721–07-5	1.11	pKa_1_ = −4.30 (basic), pKa_2_ = 13.10 (acidic)	252.27
Citalopram	CIT	59729–33-8	3.5	pKa = 9.78 (basic)	324.39
*N*-Desmethylcitalopram	DCIT	62498–67-3	3.53^a^	pKa = 10.54 (basic)	310.37
Clindamycin	CIL	18323–44-9	2.16	pKa = 7.72 (basic)	424.98
Clindamycin sulfoxide	CILS	22431–46-5	− 0.98	pKa = 11.99 (acidic)	440.98
Fexofenadine	FEX	83799–24-0	5.6	pKa_1_ = 4.04 (acidic), pKa_2_ = 9.01 (basic)	501.66
Irbesartan	IRB	138402–11-6	5.31^a^	pKa_1_ = 4.12 (basic), pKa_2_ = 7.40 (acidic)	428.53
Sulfamethoxazole	SUL	723–46-6	0.89	pKa_1_ = 1.70 (basic), pKa_2_ = 5.60 (acidic)	253.28
*N1*-Acetylsulfamethoxazole	N1AS	18607–98-2	0.51^a^	pKa_1_ = 1.85^c^ (basic), pKa_2_ = 19.66^c^ (acidic)	295.32
*N4*-Acetylsulfamethoxazole	N4AS	21312–10-7	1.21^a^	pKa_1_ = 0.78^c^ (basic), pKa_2_ = 5.68^c^ (acidic)	295.32

### Pot experiments

**Fig. 1:**
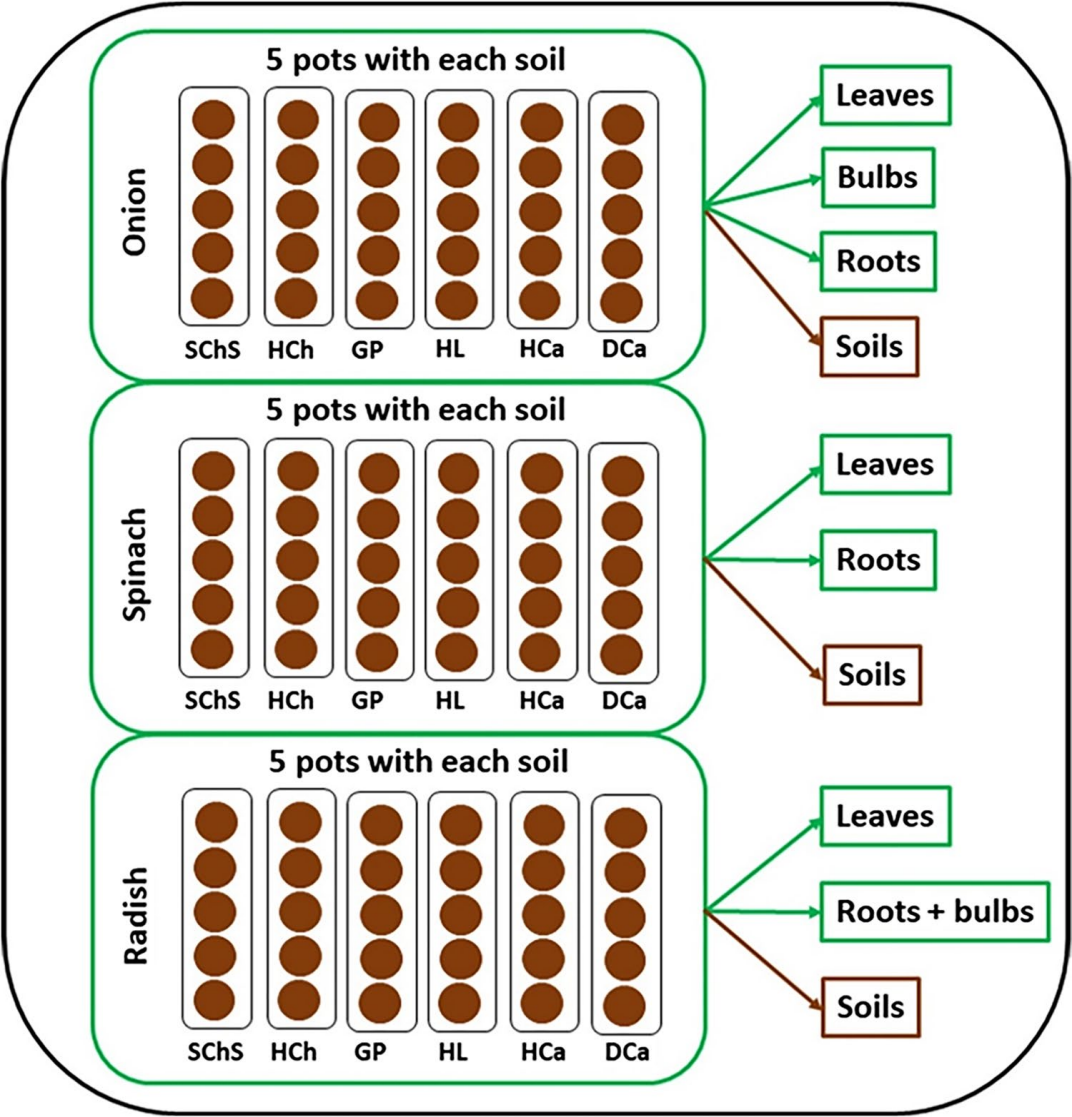
Diagrammatic representation of the pot experimental sets used in this study. Each set consists of 90 pots for studying 6 different soils (SChS, HCh, GP, HL, HCa, and DCa) and 3 plants (onion, spinach, and radish). Separate set of experiments were conducted to study the behavior of (1) carbamazepine, (2) citalopram, (3) clindamycin, (4) fexofenadine, (5) irbesartan, (6) sulfamethoxazole, and (7) all the compounds together

The experimental protocol, which is conducted in a greenhouse under controlled air temperature (20–24 °C) and humidity (30–40%), is adopted from a previous study of Kodešová et al. (2019b). During these studies, the six parent pharmaceuticals (CAR, CIT, CIL, FEX, IRB, SUL), either alone or as a mixture of all together, were applied on three selected plants (onion (*Allium cepa* L.), spinach (*Spinacea oleracea* L.), and radish (*Raphanus sativus* L.)) grown in small pots (volume: 340 cm^3^) filled with one of the six selected soils taken from the surface horizons of the following soil types: Stagnic Chernozem Siltic (SChS), Haplic Chernozem (HCh), Greyic Phaeozem (GP), Haplic Luvisol (HL), Haplic Cambisol (HCa), Dystric Cambisol (DCa). All these soil types, either all or few of them, were recently utilized for the studies of Schmidtová et al. (2020), Kodešová et al. (2019a, b, 2020), Frková et al. (2020), and other researchers (Brunetti et al. 2021; Golovko et al. 2016; Klement et al. 2020; Menacherry et al. 2022b). The physical properties of the fresh soil samples taken for this study (e.g., soil organic content, sand and clay proportions) as well as the sorption and dissipation behaviors of the selected parent pharmaceuticals on each of them were previously analyzed by Frková et al. (2020), Kodešová et al. (2020), and Schmidtová et al. (2020). The selected data from the above-mentioned studies, which included the important soil properties and the dissipation half-lives of the parent pharmaceuticals, are also presented in supplementary information (Tables S1-S2). The experiments were conducted completely under natural lighting conditions between April and June of 2017. Diagrammatic representation of a pot experimental set, which includes 90 pots to accommodate five replicates of each plant species grown in each of the selected soil types, is available in Fig. 1. Separate sets were prepared for investigating the behavior of pharmaceuticals during their individual (six sets) and combined (one set) application.

The plants were initially irrigated with tap water followed by irrigation with the pharmaceutical solutions (either alone or as a mixture of all on separate experiential sets) of an intended concentration of 1 mg L^−1^. The timeline of the irrigation process with the pharmaceuticals along with other important experimental events is listed in Table S3. After the experimental timescale, the plants were carefully recollected from each pot and divided into separate parts, such as the roots, bulb (for onion), and leaves, after necessary cleaning and freeze drying. It should be noted that the “root” of radish hereinafter referred in this manuscript also includes its bulb portions as well. Furthermore, the onions were harvested in an early growth stage (i.e., spring onion) in which both bulbs and leaves are consumable. The soils from each pot were also recollected and freeze dried. Both plant parts and soils recovered from the five replicates were combined and the new set of samples obtained (one each for soils and plant parts) were used for further analysis to evaluate their pharmaceutical contents (please see “Chemical analyses” section for more details). The dry weights of the combined plant tissues are available in Table S4. Although we took precautions to avoid the loss of molecules from our experimental environment (i.e., pots) as much as possible, the presence of drainage holes in the pots resulted the leaching of a small amount of liquids and thereby make it impossible for us to perform mass balance analysis or to calculate the parent compound load normalized concentration values (Klement et al. 2020; Kodešová et al. 2019a, b). However, the molar fractions (i.e., the proportion of a specific molecule in percentage detected on a given environment; e.g., plant tissues) and the bio-accumulation factors (i.e., BAF; a ratio obtained from the concentrations in plant tissue and the concentrations in soil measured after harvest) were calculated and used for the comparative studies (Kodešová et al. 2019a). The calculation of BAF in the case of metabolites, however, utilizes the molar masses of each associated compound. Furthermore, despite the lack of error bars, it should be noted that the individual values represented in our figures are the average of five separate replications, which enhances the reliability.

### Chemical analyses

#### Extraction of pharmaceuticals from soils and plants

A previously reported, ultrasound-based, extraction approach with two solvent mixtures was applied to analyze various compounds from soil samples (Golovko et al. 2016; Kodešová et al. 2019a, 2020). In brief, about 2 g of each soil sample after freeze-drying was placed into a 10-mL auto sampler vial. About 20 ng of internal standard was then added. The samples were then extracted with 4 mL of mixture 1 (acetonitrile/water 1/1, v/v acidified with 0.1% of formic acid) for 15 min on an ultrasonic bath (DT 255, Bandelin Electronic, Sonorex Digitec, Berlin, Germany). After carefully collecting the supernatants, the above extraction step was repeated with 4 mL of mixture 2 (acetonitrile/2-propanol/water, 3/3/4, v/v/v, acidified with 0.1% of formic acid). Both supernatants were finally combined and filtered through a syringe filter (0.45 μm, regenerated cellulose, Labicom, Olomouc, Czech Republic) before liquid chromatography-tandem mass spectrometry (LC-MS/MS) analysis.

Similarly, another well-established method by Kodešová et al. (2019a, b) has been utilized to analyze the compounds from plants. In short, 0.05 g of the freeze-dried plant samples along with 5 ng of internal standard, a stainless-steel ball, and 1 mL of extraction mixture 1 was placed in an Eppendorf tube with a safe lock. This tube is then subjected to vigorous shaking at 1800 min^−1^ (TissueLyser II, Quiagen, Germany) for 5 min. The samples were then centrifuged at 10,000 min^−1^ for 5 min (Mini spin centrifuge, Eppendorf). The supernatant solution was filtered through the previously mentioned 0.45 µm syringe filter before subjecting to LC–MS/MS analysis.

#### Analysis of pharmaceuticals and their metabolites by LC-MS/MS

The concentrations of pharmaceuticals and their metabolites prepared after the above-described steps were determined by LC–MS/MS using either an isotope dilution or an internal standard (IS) method. Analyses of plant and soil samples were conducted on a Q Exactive HF Hybrid Quadrupole-Orbitrap mass spectrometer (Thermo Fisher Scientific, San Jose, CA, USA), which utilizes a Vanquish Pump (Dionex, Germany) and a PAL RSI autosampler (CTC Analytics AG, Switzerland). The chromatographic separation of the targeted compounds has been achieved on a Thermo Scientific Hypersil Gold aQ column (50 mm × 2.1 mm × 5 μm) against a gradient elution of water and acetonitrile (both containing 0.1% formic acid). A more detailed description of the instrumental set up has been published earlier (Grabicova et al. 2018). Much more information about the analytical conditions, such as the gradient elution conditions (Table S5), m/z values, and retention times of each analyte, is available in supplementary information (Table S6). The matrix effects were corrected using a matrix-matching standard.

Moreover, the above-described analytical method for soil analyses is already validated for a wide range of compounds (including CAR, CIT, CLI, FEX, IRB, SUL, and their metabolites) and soils (including the soils used in this study) (Brunetti et al. 2021, 2022; Golovko et al. 2016; Kodešová et al. 2020; Menacherry et al. 2022b). The method used for the analysis of plant tissues is also validated for three leafy vegetables (arugula, spinach, and lamb’s lettuce), radish (Kodešová et al. 2019a, b), green pea (Klement et al. 2020), spinach (Kodešová et al. 2019a), and others (Brunetti et al. 2021, 2022). Despite this fact, the method was initially tested at a fortification level of 100 ng g^−1^ and 1000 ng g^−1^ for soils and plants, respectively (Table S7). Corrections were assumed with respect to recoveries when analyzing concentrations in soil and plant tissues. In addition, duplicates of every third sample were analyzed. The limits of quantification (LOQs) of compounds in all matrices are presented in Table S8.

## Results

### Accumulation of pharmaceuticals in plants: single application

**Fig. 2:**
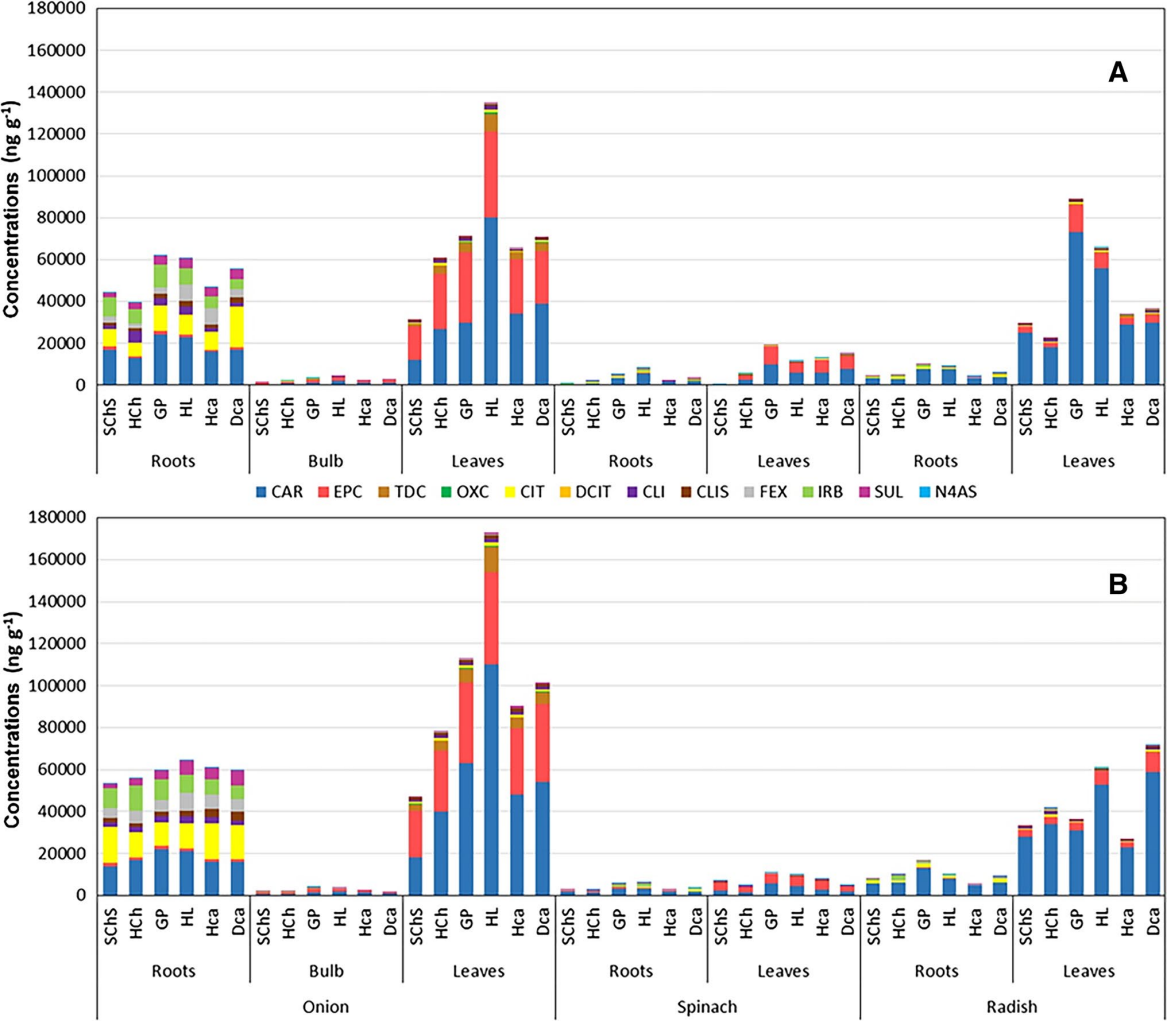
Concentration (in ng g^−1^) of pharmaceuticals and their selected metabolites detected in the roots and leaves of onion, spinach, and radish after (**A**) single and (**B**) simultaneous applications. The concentration of compounds quantified in the bulb of onion is also included. For the exact values, please refer to Tables S9 and S10

The concentration of pharmaceuticals, as well as their metabolites, accumulated on various parts of plants (e.g., such as the roots and leaves of onion, spinach, and radish; bulb of onion) grown in six selected soil types after their single application were initially measured and compared (Fig. 2). Although we provided the respective accumulation data of pharmaceuticals after their simultaneous application in Fig. 2, the presentation of the same is conducted only in a later section. Incidentally, it should be noted that the intended initial concentrations (1 mg L^−1^) of parent pharmaceutical utilized for this study are notably higher than that of their reported environmental concentrations (Brunetti et al. 2022; Golovko et al. 2014b, a; Loos et al. 2013). As an example, the maximum detected concentrations of CIT and IRB in the wastewater effluents were approximately 200 and 1800 ng/L respectively (Loos et al. 2013), which were several order of magnitudes lower than the intended initial concentration considered for this study. The use of higher concentration of pharmaceuticals, however, helped us to ensure the detection of their selected metabolites, which are otherwise unlikely from an environmentally relevant lower concentration. Moreover, although concentration-dependent changes are expected up to certain extend, it is believed that the behavior of pharmaceuticals in plants, such as their uptake, accumulation, and metabolism, may not be too different for higher tested concentrations. It is, thus, assumed that the experiments conducted even at higher initial concentrations will deliver meaningful outcomes.

Among the three plant species considered, the highest pharmaceutical accumulation was found in onion followed by radish and spinach. Moreover, the highest accumulation was detected to be in the plant leaves rather than the roots and bulb (only for onion). Irrespective of the soil type and plant part, CAR is the highest accumulated pharmaceutical, which is in well agreement with previous studies (Barreales-Suárez et al. 2021; Ben Mordechay et al. 2022; Klement et al. 2020; Kodešová et al. 2019b; Pérez et al. 2022). While a noticeable accumulation of other parent pharmaceuticals is observed in the onion roots, the same on other plants seems less significant. It is worth noting that the recorded concentration of CIT in onion roots grown in a particular soil type (i.e., DCa) is more or less similar to that of CAR. Furthermore, it is interesting to note that an appreciable amount of EPC, a primary metabolite of CAR, is also measured from the plant leaves. The accumulation of this specific metabolite, which reported to have “potential genotoxic effects” by Meffe et al. (2021), on several plant leaves (e.g., carrots, sweet potatoes, lettuce, etc.) with concentrations that are equal or even higher than that of its parent compound (i.e., CAR) is previously noticed (Malchi et al. 2014; Riemenschneider et al. 2016). Another metabolite of CAR, TDC, was also measured in some plant leaves (e.g., onion) with significant amounts. The combined concentration of CAR and its metabolites (except DHC, which is not detected in plant tissues throughout this study), marked the most majority of pharmaceutical accumulation recorded in the case of all the plant leaves.

The total accumulated concentration of molecules (i.e., either a parent pharmaceutical or a metabolite) in an entire plant species was calculated for both applications (i.e., single or simultaneous) as the sum of individual concentrations in each plant tissues multiplied by their dry weights, divided by the total dry weight of all the tissues (i.e., total dry weight of plants). The results as shown in Table S11 revealed the maximum accumulation of CAR on onion plants (root + bulb + leaves) grown in HL (~ 38,000 ng g^−1^) followed by DCa (~ 18,000 ng g^−1^), HCa (~ 17,000 ng g^−1^), HCh (~ 15,000 ng g^−1^), GP (~ 17,000 ng g^−1^), and SChS (~ 7500 ng g^−1^). This trend is slightly different in the case of other two plants (i.e., spinach and radish; root + leaves), where the highest accumulations of this molecule are observed in GP (~ 7500 ng g^−1^ for radish and ~ 43,000 ng g^−1^ for spinach) followed by HL (~ 36,000 ng g^−1^ for radish and ~ 5700 ng g^−1^ for spinach), DCa (~ 18,000 ng g^−1^ for radish and ~ 5700 ng g^−1^ for spinach), and HCa (~ 17,300 ng g^−1^ for radish and ~ 4300 ng g^−1^ for spinach). Analogous to onion, the lowest values of CAR were recorded in the case of SChS (~ 15,200 ng g^−1^ for radish and ~ 160 ng g^−1^ for spinach) and HCh (~ 11,300 ng g^−1^ for radish and ~ 1800 ng g^−1^ for spinach). Irrespective of the plant species, the highest CIT accumulation was recorded in DCa (~ 3300 ng g^−1^ for onion; ~ 270 ng g^−1^ for spinach; ~ 700 ng g^−1^ for radish). Appreciable amounts of IRB (GP; ~ 2100 ng g^−1^) and FEX (HL; ~ 1900 ng g^−1^) were also recorded in the case of onion, which is happened to be mostly in roots. The latter compound, IRB, has been previously demonstrated to be accumulated on aquatic mosses receiving a higher dose of that molecule (Sossey Alaoui et al. 2021). Furthermore, the onion plants grown in HL recorded the highest CLI (~ 1500 ng g^−1^) and SUL (~ 1000 ng g^−1^) accumulations. Considering the faster degradation behavior (Kodešová et al. 2016, 2020) and lower dissipation half-lives (Table S2) of these molecules, these kinds of lower accumulation are not surprising. The pharmaceuticals accumulated in the remaining cases seem less significant and thereby excluded from presenting their exact values in this report.

Among the metabolites, the significant accumulation was mostly happened in the case of EPC (~ 19,000 ng g^−1^ for onion — HL; ~ 6300 ng g^−1^ for spinach — GP; ~ 7600 ng g^−1^ for radish — GP) followed by TDC (~ 4000 ng g^−1^ for onion — HL; ~ 80 ng g^−1^ for spinach — HCa and DCa; ~ 270 ng g^−1^ for radish — GP), and CLIS (~ 930 ng g^−1^ for onion — HL; ~ 260 ng g^−1^ for spinach — DCa; ~ 460 ng g^−1^ for radish — GP). The concentration of remaining metabolites accumulated into the plant species seems either low (e.g., OXC, DCIT, N4AS) or completely insignificant (e.g., DHC and N1AS). It is important to note that the majority of metabolites (e.g., EPC and TDC) detected in an appreciable concentration is mainly accumulated into the edible plant parts.

The above-explained data, on the other hand, considered the total amount of pharmaceuticals accumulated on all over the plants. Specifically evaluating the accumulation of these compounds into the edible pant parts, such as the leaves and bulb (onion), is also important. The percentage accumulation of the parent pharmaceuticals and their metabolites after both the single (Table S12) and simultaneous (Table S13) applications were, therefore, calculated and compared. It is estimated that the percentage of CAR accumulated into the edible part of onion (i.e., the bulb and leaves) is varied from 70% (SChS) to 90% (HL). The percentage of CAR accumulated into the leaves of spinach (65% — HL to 89% — DCa) and radish (88% — HCh to 92% — GP) also recorded impressive values. This observation justifies the recent study of Kreuzig et al. (2021) reporting the accumulation of CAR into the edible parts of lettuce (120 μg kg^−1^), which is nearly double in concentration to that of its accumulation in roots (69.5 μg kg^−1^). More or less similar observations were also reported by Kodešová et al. (2019a, b). This is, however, significantly different to that of other CECs, such as acesulfame, diclofenac (Kreuzig et al. 2021), metoprolol, sertraline, miconazole, CIT (Kodešová et al. 2019a), atenolol, and SUL (Kodešová et al. 2019b), where the accumulation is either less prominent (e.g., acesulfame) or insignificant (e.g., diclofenac) in the case of the edible plant parts. Our results also justified the above observation as the percentage accumulations of other pharmaceuticals, such as CIT (11–33%), CLI (38–46%), and SUL (9–18%), on the onion leaves were less significant to that of CAR. Upon comparing the exact values, the accumulation of pharmaceuticals into the edible parts of plants seems to be significant mainly in the case of CAR. Furthermore, it is worth mentioning that the exact concentration of that molecule (i.e., CAR) accumulated into the edible parts of onion is several times higher than that of the one in the case of spinach and radish. By considering the limited accumulation of other pharmaceuticals on radish and spinach plants as shown in Fig. 2 (please see Tables S9 and S10 for exact values), we restricted discussing the respective data from this report. The use of radish roots for dietary purposes, on the other hand, demanded more attention on the pharmaceutical accumulation behavior of this plant. As a result, the noticeable amounts of CAR (> 7500 ng g^−1^ for GP and HL; > 3000 ng g^−1^ for SChS, HCh, HCa, and DCa), IRB (> 900 ng g^−1^ for GP and HCh; > 600 ng g^−1^ for SChS and DCa), SUL (> 300 ng g^−1^ for HCa, GP, and HL), and CIT (~ 1000 ng g^−1^ for DCa; > 600 ng g^−1^ for GP and HCh) accumulated into the radish roots were equally important.

As we already discussed, the accumulation of some metabolites was mainly occurred in the edible plant parts. For example, the accumulation of EPC, the metabolite which is quantified in amount as equal to CAR in our work and other previous studies (Kodešová et al. 2019a; Malchi et al. 2014; Meffe et al. 2021; Riemenschneider et al. 2016), into the leaves of onion, spinach, and radish often crosses 90% of its total accumulation. Another important metabolite, TDC, was almost exclusively accumulated into the edible plant parts for all the tested plant species. The amount of CAR metabolites found to be the least in radish leaves in comparison with onion and spinach leaves. This fact is in good agreement with the previous study of Kodešová et al. (2019b) reporting the less efficiency of plants from the *brassicaceae* family to metabolize that molecule. The accumulation of CLIS and OXC into the edible plant parts also crosses 90% in some cases. The exact concentration of these metabolites is, however, less significant than that of EPC. It is worth mentioning that the amount of EPC accumulated into the radish roots, the edible part of this plant species, also marked appreciable amounts (> 200 ng g^−1^ for GP and HL).

### Pharmaceuticals remained in the soils

**Fig. 3:**
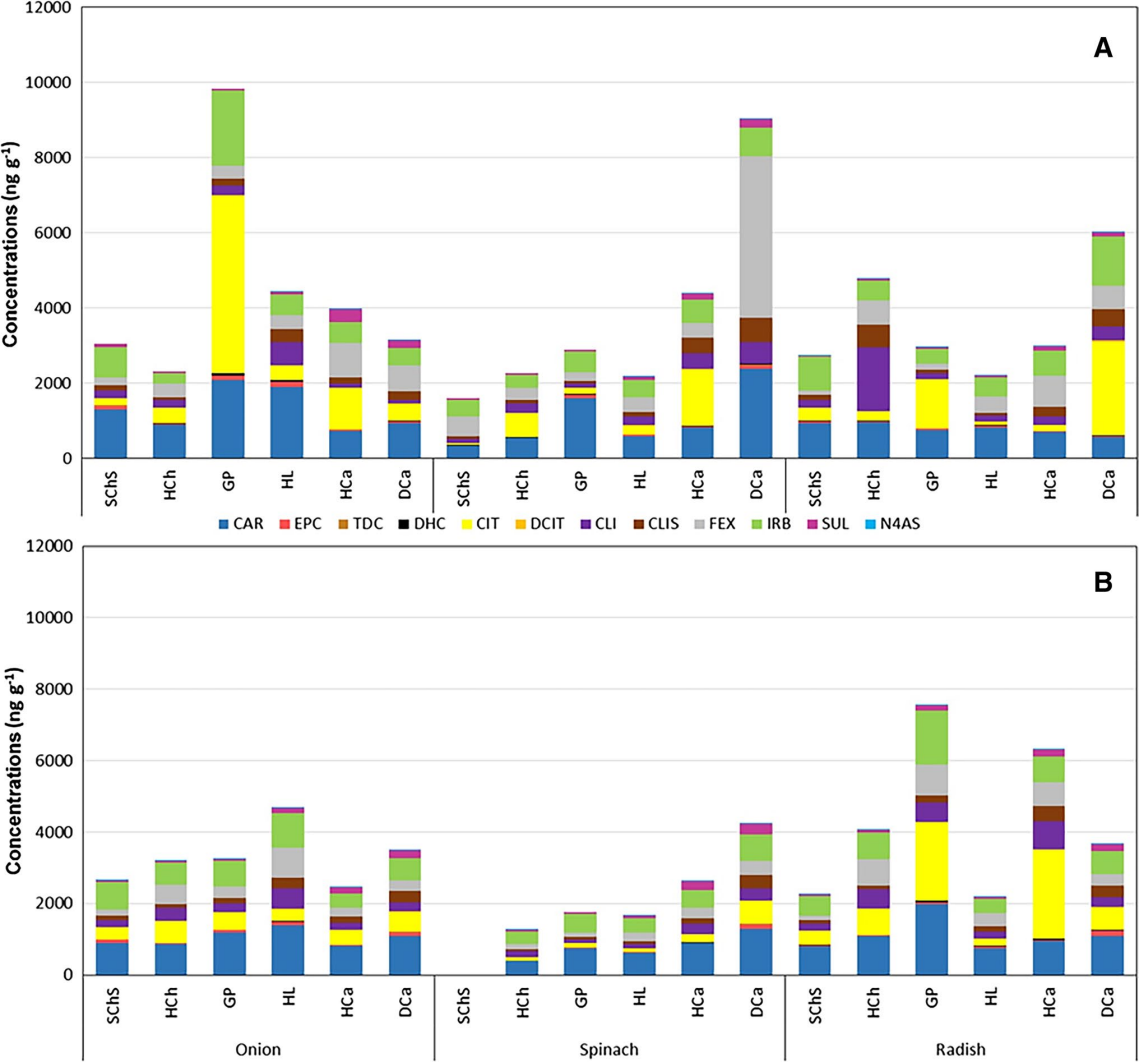
Concentration (in ng g^−1^) of pharmaceuticals and their selected metabolites remained in the soil samples after the (**A**) single and (**B**) simultaneous applications. The remaining concentrations of pharmaceuticals after their multiple application in soil SChS are excluded due to cross-contamination of samples while its processing. For the exact values, please refer to Tables S14 and S15

The remaining concentrations of parent pharmaceuticals in various soil samples after the above-mentioned experiment were also measured and compared in Fig. 3. It should be noted that the amount recovered from the soil samples may not directly represents the remaining amount of pharmaceuticals after accumulation by the plant species. An important reason behind this assumption is the possible dissipation/metabolism of the parent pharmaceuticals within the soil environment, i.e., before their accumulation by plant roots. Furthermore, the loss of pharmaceuticals from our experimental environment (i.e., pots) should also been considered. The concentrations of the selected metabolites determined from the same soil samples are also included in Fig. 3. Although reaching clear assessments from Fig. 3 are exceedingly difficult due to the above-mentioned concerns, it is expected that the results obtained from these experiments will help to understand this scenario much better. The exact values of each pharmaceutical and metabolites detected after their single (Table S14) and simultaneous (Table S15) applications can be found in the supplementary information.

It is well clear from Fig. 3A that the concentration of pharmaceuticals recovered after their single application was maximum in the case of soils GP (onion) and DCa (spinach and radish). This trend is in a reasonable agreement with the accumulation behavior of pharmaceuticals as depicted in Fig. 2. For example, in contrast with the highest accumulation of pharmaceuticals in onion plants grown in HL, the remaining concentration of these molecules recovered from this soil is significantly lower. Similarly, the higher amount of pharmaceuticals recovered from the soil type DCa is in well agreement with the fact that the accumulation level of these molecules is not the best for spinach and radish grown in that soil. Furthermore, the composition of pharmaceuticals seems significantly varied in different soil types. It is well clear that the highest recovered pharmaceutical in the case of onion (soil: GP) and radish (soil: DCa) is CIT, which is in well agreement with the highest dissipation half-lives reported for this molecule (Kodešová et al. 2020). The highest recovered pharmaceutical in the case of spinach is, however, FEX (soil: DCa). It is worth noting that, despite the efficient accumulation of this molecule into plant tissues, the amount of CAR recovered from the soil environment is still significant in most cases (Fig. 3). This fact clearly demonstrated the stability of this molecule within the soil environment as clearly noticed by previous researchers (Kodešová et al. 2020; Menacherry et al. 2022b). The results obtained after the simultaneous application of these pharmaceuticals are presented in a later section.

### Molar fraction and bio-accumulation factor of pharmaceuticals

**Fig. 4:**
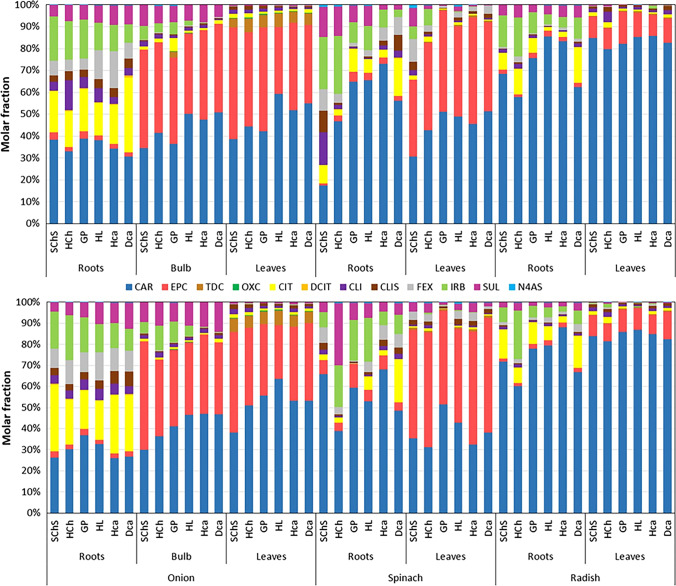
Molar fraction of pharmaceuticals and their metabolites in plants after their (top) single and (bottom) simultaneous applications

In order to explore this important scenario in more detail, the molar fractions and bio-accumulation factors (BAF) of each pharmaceutical were calculated. The calculated molar fractions of molecules for each plant tissues grown in the six selected soil types are depicted in Fig. 4. Similar to the previous sections, the results related to the single application of pharmaceuticals were only presented in this section. The same for the simultaneous application is available in next section.

The results as shown in Fig. 4 clearly revealed the efficient accumulation of CAR and its metabolites in the leaves of all the selected plants, which is in well agreement with Fig. 2 as well as the previous studies by Kodešová et al. (2019a, b) and Klement et al. (2020). More specifically, the combined molar fractions of CAR and their primary metabolites (EPC and TDC) contributed the most majority of pharmaceutical accumulation in the case of onion (bulb: ~ 80–90%; leaves: ~ 93–96%), spinach (leaves: ~ 67–97%), and radish (leaves: ~ 89–96%). It is worth noting that the molar fraction of EPC in many cases crosses the one for CAR. Appreciable portions of TDC were mainly detected in onion leaves. The molar fraction of CAR metabolites was less significant in the plant roots, irrespective of the soil and plant type. Despite this fact (i.e., the less significant metabolite fractions), the individual fraction of CAR in the plant roots seems to be significant. The molar fractions of other pharmaceuticals, such as CIT, IRB, SUL, FEX, and CLI, are also appreciable in the case of plant roots.

**Fig. 5:**
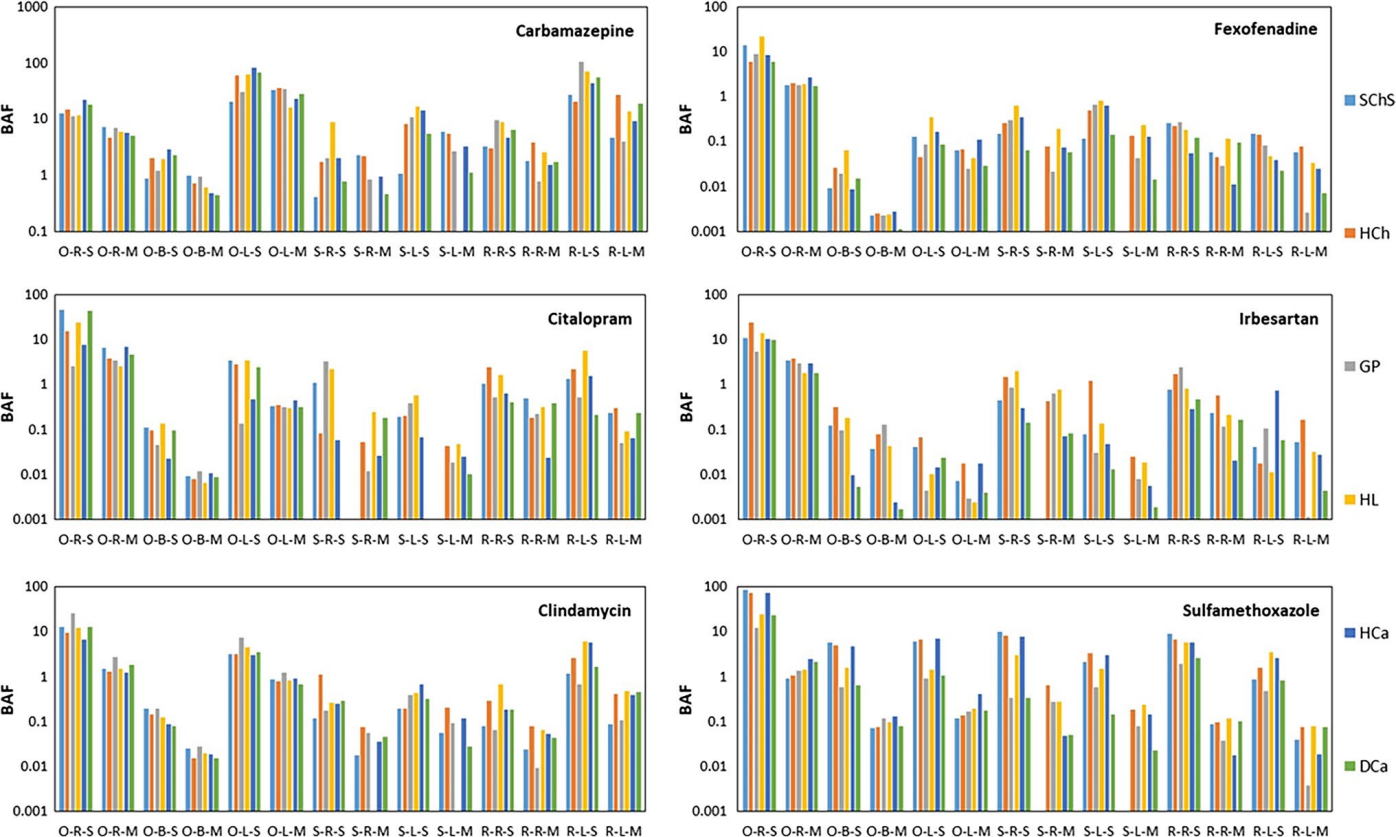
Bio-accumulation factors of parent pharmaceuticals calculated for different plants species (O, onion; S, spinach; R, radish), tissues (R, roots; B, bulb; L, leaves), soils (from left to right; light blue, SChS; orange, HCh; grey, GP; yellow, HL; dark blue, HCa; green, DCa) and application modes (S, single; M, mixture of all 6 compounds). For the exact values, please refer to Tables S16 and S17

The BAF calculated for individual pharmaceuticals, however, showed distinguishable trends (Fig. 5). The resulting BAF values for CAR clearly showed that the most majority of this molecule (and their metabolites) was accumulated largely into the plant leaves (lowest ~ 20 (soil: SChS); highest ~ 81 (soil: HCa)) than the roots (lowest ~ 11 (soil: GP); highest ~ 21 (soil: HCa)) and bulbs (lowest < 1 (soil: SChS); highest ~ 2.8 (soil: HCa) — only for onion). Furthermore, analogs to the accumulation trend as shown in Fig. 2, BAF of onion and radish leaves (lowest ~ 20 (soil: HCh); highest ~ 107 (soil: GP)) seems to be more superior to that of the one calculated for spinach leaves (lowest ~ 1 (soil: SChS); highest ~ 16 (soil: HL)). Very high BAF value was also observed in the case of SUL (onion), but with the majority of accumulation happened in the case of plant roots (lowest ~ 12 (soil: HCa); highest ~ 81 (soil: GP)). Except for certain cases (mainly for onion root), the majority of the other pharmaceuticals does not show appreciable BAF values (BAF < 10) in which many of them were even lower than 1 representing an insignificant accumulation in comparison with the medium in which the uptake happened (Gworek et al. 2021). The calculations made for the simultaneous application of all the tested pharmaceuticals were also provided in Fig. 5, while the respective discussion available in next section. The exact BAF values calculated for each pharmaceutical (including separate plant tissues) grown in the selected soil types after single (Table S16) and simultaneous (Table S17) applications are available in the supplementary information.

### Accumulation of pharmaceuticals in plants after simultaneous application

The presence of other chemical species, which are plenty in natural soil environment, variably impacts the fate (e.g., metabolism) and the uptake of a specific pharmaceutical as evident from previous studies (Beltrán et al. 2021; Khalaf et al. 2022; Kodešová et al. 2020; Liang et al. 2019). It is, thus, important to evaluate the uptake of these pharmaceuticals in a mixed environment to better correlate these results with a more realistic nature. In order to address this issue, an additional set of pot experiments were conducted in which all the pharmaceuticals were applied together.

It is well clear from Fig. 2B (please see Tables S10 for exact values) that the behavior of pharmaceutical uptake and translocation follows a general trend, irrespective of their mode of application (i.e., single or mixed). The extent of accumulation is, on the other hand, quantitatively different in the case of mixed application mode. As an example, the accumulation of CAR in the leaves of onion planted in HL seems more than 25% higher when this molecule was applied along with other pharmaceuticals (i.e., mixed application mode). However, the increase in the accumulation of its primary metabolite, EPC, was very little (~ 6%). The accumulations of this molecule on the spinach and radish leaves are markedly suppressed (> 40% for spinach and > 55% for radish; soil: GP) when applied together. Furthermore, the highest accumulation of CAR in radish leaves after the mixed application of pharmaceuticals seems more significant in the case of soil type DCa, which is in contrary to that of the single application mode. The general trend of pharmaceutical accumulations includes (i) higher accumulation of pharmaceuticals by onion than radish and spinach, (ii) markedly higher accumulation of pharmaceuticals into the plant leaves than their roots, and (iii) identification of CAR as the highest accumulated molecule in all the tested plants and soil types. This assessment is further verified by comparing the molar fraction of pharmaceuticals between the single and mixed application modes (Fig. 4) exhibiting very similar patterns, except the minor changes due to the above-mentioned differences. A graphical comparison of all the parent pharmaceuticals and their metabolites measured in the case of plant species grown in different soil types after their single and simultaneous applications is provided in the supplementary information (Fig. S1). Although the coefficients of determination (R^2^) describing the goodness of fitting is not excellent in some cases, the similarities between the single and simultaneous application results clearly obey the above-mentioned general accumulation trend.

Furthermore, other than the consistently lower values recorded after the simultaneous application of pharmaceuticals as shown in Fig. 5, the BAF calculations also follow a comparable trend to that of the single application. Minor differences were also observed in the remaining concentration of pharmaceuticals in soil as well (Fig. 3B; please see Tables S15 for exact values), indicating either an enhancement in their accumulation (e.g., onion leaves) or their faster dissipation after applying them simultaneously as already noticed by various researchers (Khalaf et al. 2022; Menacherry et al. 2022b; Papaioannou et al. 2019; Styrishave et al. 2011; Zheng and Guo 2021). By considering the differences in the BAF calculations between the single and simultaneous application (which accounts the possible degradation of parent pharmaceuticals in the soil environment), such changes are well anticipated.

## Discussion

It is already mentioned the impact of physical and chemical parameters of pharmaceuticals on their uptake and translocation on a specific plant species grown in a given soil type (Brunetti et al. 2021, 2022; Collins et al. 2006, 2011; Klement et al. 2020; Kodešová et al. 2019b; Kurade et al. 2021; Miller et al. 2016). Additionally, the stability of an individual pharmaceutical on a given soil type, which in-turn is associated with several factors, such as the soil–molecule interactions, efficiency of degradation pathways, and various other environmental factors (Diez 2010), is also expected to influence their uptake. By considering the established relationship between the speciation of pharmaceuticals (i.e., neutral, anionic, or cationic) on its dissipation within the soil compartments, it is expected that the molecule with higher stability and lower dissipation characteristics will be more susceptible to plant uptake and translocation through the transpiration stream (Diez 2010; Koba et al. 2016; Kodešová et al. 2015, 2016, 2020; Menacherry et al. 2022b).

Systematic studies on the transfer of pharmaceuticals existing in soils, either adsorbed on the soil solids or dissolved in the soil pore water, to plant are recently available (Christou et al. 2019; Keerthanan et al. 2021; Miller et al. 2016; Zheng and Guo 2021). Molecules with a moderate molecular size (< 500 g mol^−1^) might be more accessible to the soil pore water and can easily be carried to the root epidermis, which either results their accumulation on the root surface or the eventual crossing of cortex and endodermis to reach the root tissues by one or more of the potential translocation pathways, such as the apoplastic, symplastic, or the transmembrane pathway (Christou et al. 2019; Miller et al. 2016; Zheng and Guo 2021). Those molecules managed to enter the root tissues might be further translocated to other plant parts primarily by transpiration and diffusion processes (Zheng and Guo 2021). Neutral molecules, such as CAR (identified as a persistent, moderately mobile, and non-biodegradable) (Bueno et al. 2022; Kodešová et al. 2020; Menacherry et al. 2022b), staying longer in the soil environment are known to cross the cell membranes without much difficulties (Christou et al. 2019). As a result, the chances of these molecules (e.g., CAR) to be taken up and translocated to the edible parts of plants are recognized as extremely efficient in the case of several plants (Ben Mordechay et al. 2022; Klement et al. 2020; Kodešová et al. 2019b; Pérez et al. 2022). Our results are in well agreement with the above statement as an efficient uptake and translocation of CAR by all the selected plant roots is observed (Fig. 2). A recent study by Pérez et al. (2022) on hydroponically grown corn clearly established a strong relationship between the lipophilicity (LogK_ow_) and ionization nature (speciation) of organic molecules towards their uptake and translocation to the plant edible parts. While the neutral nature of CAR (100% neutral in most soil environments) ensure its uptake by plant roots, the low value of LogK_ow_ (~ 2.77) accelerates its translocation between the plant cells (Pérez et al. 2022).

Among the plant species, the accumulations of pharmaceuticals are lowest for spinach (Fig. 2), which is in reasonable agreement with our previous study comparing the uptake and accumulation of three pharmaceuticals (includes CAR and SUL) in three soil types (includes HCh and HCa) by four plant species (includes spinach and radish) (Kodešová et al. 2019b). Furthermore, the plants grown in sandy soils, soils with low clay proportion, or soils containing low organic contents have higher potential to uptake organic molecules (Christou et al. 2019; Zheng and Guo 2021). As an example, the promising accumulation of CAR into several plants (maximum up to 30 ng g^−1^) seems to be reduced in soils containing more organic contents (Ben Mordechay et al. 2022). Among the tested soil types, the percentage of organic contents (Table S1) is minimum in the case of HL (1.06%) followed by GP (1.36%), HCh (1.75%), and HCa (1.85%) (Kodešová et al. 2020; Schmidtová et al. 2020). The soil organic content values reported on another work of Kodešová et al. (2019a) also follow a similar trend except for a slightly lower value for HCa (1.51) than HCh (1.79). This trend is in well agreement with the accumulation behavior of pharmaceuticals as shown in Fig. 2, especially in the case of onion leaves; the plant compartment in which the highest accumulation observed. The accumulation trend in spinach and radish leaves was also in a reasonable agreement with the above-mentioned logic except in the case of DCa (soil organic content: 2.23%, Table S1) where a significant accumulation rate is observed for both the cases. A likely reason for this observation is the low clay (16.78%) and high sand (28.93%) proportions of that soil, which might overpower the drawbacks of high organic content (2.05) (Kodešová et al. 2020; Schmidtová et al. 2020). The lowest accumulation of pharmaceuticals is observed in the case of plants grown in soil type with highest organic content (2.89%) and a medium sand (27.2%) and clay (20.7%) proportions (i.e., SChS) (Kodešová et al. 2020; Schmidtová et al. 2020). The only exception to this scenario is observed in the case of radish leaves where the total accumulation of pharmaceuticals is slightly lower for HCh, a soil with an average organic content of 1.75%. This difference is, however, not surprising by considering the negligible sand (5.4%) and the higher clay (36.5%) proportions of this soil (Kodešová et al. 2020; Schmidtová et al. 2020), which are already recognized to oppose the accumulation behavior (Christou et al. 2019; Zheng and Guo 2021).

The better stability in soil, lower molecular size (MW 236.27 g/mol), neutral speciation, and the favorable LogK_ow_ explains the efficient accumulation of CAR observed in the present study (Bueno et al. 2022; Kodešová et al. 2020; Menacherry et al. 2022b). All other pharmaceuticals selected for this study, however, possess ionic nature (mostly cationic, but neutral and zwitterionic characteristics by FEX and IRB) up to certain extends. The LogK_ow_ of most of these drugs, except CLI and SUL, is marginally higher than that of CAR, with values over 5 for IRB and FEX (Table 1). It has been recently noticed that the CECs of higher LogK_ow_ values (e.g., triclosan and gemfibrozil) may not be translocated with the transpiration stream and thereby limits their presence only into plant roots (Pérez et al. 2022). Our results as presented in Figs. 1 and 4 are in full support to this proposal as the accumulation of the two compounds with higher LogK_ow_ of more than 5 (i.e., IRB and FEX) only occurred in the plant roots, with insignificant accumulation in the bulb (only for onion) and leaves. Among the remaining pharmaceuticals, CLI and SUL exhibit lower LogK_ow_ value to that of CAR. However, the rapid degradation of these molecules within the soil environment makes them less available for uptake by plant roots (Koba et al. 2017; Kodešová et al. 2016, 2020). Furthermore, the uptake of the majority of these drugs, except CAR, is demonstrated as very little to moderate in the case of lamb’s lettuce, spinach, radish, green pea, and other plants (Beltrán et al. 2021; Klement et al. 2020; Kodešová et al. 2019a, b). The half-lives of most drugs, except CAR and CIT, tested for our studies were very lower, in which SUL being the one with the least stability in all the tested soil types (Table S2). As a result, the insignificant uptake of SUL, which hardly managed to reach up to the roots of certain plants (e.g., onion; Fig. 2), is very much logical. The partially neutral nature and the comparable LogK_ow_ (to that of CAR, Table 1), on the other hand, resulted the noticeable accumulation of CLI on the onion and radish leaves. Similar observation has previously been reported in the case of atrazine, a molecule with a partial cationic characteristics, but possess a comparable LogK_ow_ of 2.20 (Pérez et al. 2022). Despite the restricted uptake of this molecule due to its partial cationic nature, this molecule seems to be translocated very efficiently from the root to the edible parts of corn grown in hydroponic conditions.

The metabolism of pharmaceuticals often led to the generation of molecules with more hydrophilic nature and less formula weight (Zheng and Guo 2021). Both of these processes have the potentials to affect the uptake and translocation of organic molecules as evident from the previous studies (Christou et al. 2019; Chuang et al. 2019; Zheng and Guo 2021). Furthermore, the metabolites or degradation products of many pharmaceuticals are structurally similar (Koba et al. 2016, 2017; Menacherry et al. 2022a, b; Menachery et al. 2018; Sreekanth et al. 2014; Sunil Paul et al. 2014; Zheng and Guo 2021) and known to exhibit equal or even more toxicity (e.g., cephalosporin) (Ribeiro et al. 2018; Zheng and Guo 2021) to that of the parent pharmaceuticals. As a result, degradation of the parent pharmaceuticals within the experimental environment likely influences the uptake/accumulation behavior and thereby demands more discussions. Among the four metabolites of CAR, three of them (i.e., EPC, TDC, and OXC) exhibiting values of LogK_ow_ much lower than that of the parent molecule (Table 1) seem to be accumulated in almost every plant leaf (Table S9). The reaming one, DHC, is found to be generated in the soil environment, but failed to reach the plant parts, has a higher LogK_ow_ (2.46) than CAR and the other metabolites (Table 1). The remaining metabolites found to be taken up by the plant roots are DCIT and CILS, in which the latter one is accumulated into the edible plant parts in noticeable amounts. The p*K*_a_ of CLIS, which is marginally higher than that of CIL (Table 1), is expected to make this molecule more neutral in our soil environment and thereby enhances its translocation into the edible part of plants. Furthermore, the LogK_ow_ of this metabolite is significantly lower than that of the parent molecule, which also expected to enhance the translocation of molecules within the plant compartments (Pérez et al. 2022). Both p*K*_a_ and LogK_ow_ values of DCIT are, on the other hand, not too different to that of its parent molecule to induce a similar effect. Another important drug, exhibiting a lower LogK_ow_ value, which experimentally demonstrated to metabolize very efficiently within the plant tissues (e.g., carrot (Wu et al. 2016) and radish (Li et al. 2018)) is SUL. It should be noted that the above-cited studies, however, utilizes either hydroponically grown plants (Li et al. 2018) or the direct exposure of pharmaceutical with the plant cell cultures (Wu et al. 2016). Both cases do not account the possible degradation of the parent molecule (i.e., SUL) induced by the microbial communities within the soil environment, which is recognized as an important factor from the measured half-life’s values (Table S2). Hence, the negligible accumulations of SUL and its metabolites are most likely due to the fast degradation of the parent molecule as clearly visible in Fig. 3. The metabolism of a parent pharmaceutical is expected to occur both in the soil environment (i.e., before uptake) by various mechanisms (e.g., microbial degradation) (Biel-Maeso et al. 2019; Diez 2010; Kodešová et al. 2020), and within the plant leaves (i.e., after uptake) as a consequence of enzymatic biotransformation (Kurade et al. 2021; Ravichandran and Philip 2021). An efficient metabolism of one of the parent drug, CAR, within the plant cells was previously reported by Ravichandran and Philip (2021). Most of the metabolites that are accumulated into plant cells (especially leaves; Fig. 2) are also detected in the soil samples (Fig. 3), except the OXC, clearly revealing the fact that the generation of this molecule happened only in plant cells via enzymatic mechanisms. Similarly, another CAR metabolite (DHC) generated in soil samples does not be detected in the plant cells, suggesting its exclusive generation in soils.

The dissipation (Kodešová et al. 2020; Liang et al. 2019; Menacherry et al. 2022b) and uptake (Khalaf et al. 2022; Papaioannou et al. 2019; Zheng and Guo 2021) of pharmaceutical are known to be influenced by other molecules existing in the reaction environment. This is mainly due to the increased organic load, which potentially affect the growth of microbial communities in the soil environment. Previous studies on the concentration-dependent dissipation of several pharmaceuticals revealed the fact that the above-mentioned increase in the organic load variably affects the dissipation rate (Liang et al. 2019; Lonappan et al. 2017; Zhang et al. 2013). It includes a negative effect on the dissipation of pharmaceuticals due to the microbial inhibition caused by high chemical loading levels (Shen et al. 2018; Thiele-Bruhn and Beck 2005; Zhang et al. 2013). On the other hand, an optimal organic load provides more nutritional sources for the growth of soil microbes, which eventually increases the dissipation of pharmaceuticals by enhancing biotic pathways (Menacherry et al. 2022b; Shen et al. 2018). Similar situation, i.e., either a positive or negative impact on the dissipation of pharmaceuticals, might also been expected upon their simultaneous applications. This will impact the uptake and accumulation behavior of parent pharmaceuticals, as well as their metabolites, as already demonstrated in the case of tramadol, clarithromycin, metoprolol, CAR, and more (Khalaf et al. 2022; Papaioannou et al. 2019; Zheng and Guo 2021). A comparison of the accumulated pharmaceutical concentrations after their single and simultaneous application modes clearly revealed distinguishable trends, which are already mentioned earlier (please see previous section). Although a complete elucidation of this complex scenario seems exceedingly difficult and demands more experimental efforts, which are beyond the scope of this study. However, it is expected that the above-explained scenarios likely contributed significantly to the behavior of pharmaceutical dissipation and accumulation observed in the present case.

The accumulation of pharmaceuticals onto the plant tissues, especially on the leaves and other edible parts, might have serious impacts on both the health of plant itself and to other living species consuming them as a part of food chain. The evidences to support the first assumption (i.e., the impact on plant health) is well recognized in the case of lettuce, where the exposure of various medications, such as nevirapine (Akenga et al. 2021) and acetaminophen (Leitão et al. 2021), induces noticeable impacts. Some pharmaceuticals (including CAR and IRB) even disturb the chlorophyll content and redox status of aquatic mosses, but without effecting the growth of plant species that much (Sossey Alaoui et al. 2021). Furthermore, it is expected that this impact should be more significant in the case of pharmaceuticals/metabolites exhibiting higher stability and more susceptibility towards the root uptake (and translocated between the plant cells). Therefore, the accumulation of some parent pharmaceuticals (e.g., CAR) and metabolites (e.g., EPC, TDC, CLIS) on the edible plant parts in significant amounts might have serious impacts on the health of humankind and other biota consuming such plants as a part of food chain.

## Conclusions

The uptake and accumulation of six widely used pharmaceutical compounds (either along or as a mixture of all together) of different therapeutic uses (carbamazepine, citalopram, clindamycin, sulfamethoxazole, fexofenadine, and irbesartan) by three different plant species (onion, spinach, and radish) grown in greenhouse conditions on six different soil types (Stagnic Chernozem Siltic, Haplic Chernozem, Greyic Phaeozem, Haplic Luvisol, Haplic Cambisol, Dystric Cambisol) were investigated and compared. Pharmaceuticals with neutral speciation (e.g., carbamazepine) seem to be accumulated preferentially into the plant leaves, irrespective of the plant origin and soil composition. While the highest accumulation of pharmaceuticals occurred on the plant leaves (onion > radish > spinach), the same for the roots (and bulb of onion) are less significant, except for the roots of onion. The significant stability in soil environment, the susceptibility to be taken up by the plant roots, and the efficient transport of carbamazepine and some of their primary metabolites, such as carbamazepine 10,11-epoxide and *rac trans*-10,11-dihydro-10,11-dihydroxy carbamazepine, into the leaves of tested plant species are alarming. Clindamycin sulfoxide, a primary metabolite of clindamycin, is also managed to reach the plant leaves in appreciable amounts, which is significantly higher to that of its parent form. It will eventually increase the exposure of living species consuming (including humans) these plants as a part of their food habit. The exceptional accumulation of pharmaceuticals (e.g., carbamazepine) on onion and radish, rather than spinach, is also interesting. Furthermore, our results clearly revealed the exclusive role of enzymatic mechanisms behind the generation of some metabolites (e.g., oxcarbazepine) within the plant cells, whereas some of them (e.g., 10,11-dihydrocarbamazepine) seem to be generated only in the soil environment. These kinds of studies pointing towards the possible health risks caused by the accumulation of bioactive molecules, such as the pharmaceuticals, into the plant cells are, therefore, very relevant.

## Supplementary Information

Below is the link to the electronic supplementary material.

Supplementary file1 (DOCX 524 KB)

## Data Availability

Not applicable.
