# Supplementary material for: Selective accumulation of pharmaceutical residues from 6 different soils by plants: a comparative study on onion, radish, and spinach
Source: Environ Sci Pollut Res Int. 2023 Mar 4;30(18):54160–76. doi: 10.1007/s11356-023-26102-5 (PMC10119051; doi:10.1007/s11356-023-26102-5)
Supplement: Supplementary file 1 — Supplementary file1 (DOCX 524 KB) [file 11356_2023_26102_MOESM1_ESM.docx]

**Selective accumulation of pharmaceutical residues from 6 different soils by plants: A comparative study on onion, radish, and spinach**

Sunil Paul M. Menacherry^1,^*, Radka Kodešová^1,^*, Helena Švecová^2^, Aleš Klement^1^, Miroslav Fér^1^, Antonín Nikodem^1^, Roman Grabic^2^

^1^ Czech University of Life Sciences Prague, Faculty of Agrobiology, Food and Natural Resources, Dept. of Soil Science and Soil Protection, Kamýcká 129, CZ-16500 Prague 6, Czech Republic

^2^ University of South Bohemia in České Budějovice, Faculty of Fisheries and Protection of Waters, South Bohemian Research Center of Aquaculture and Biodiversity of Hydrocenoses, Zátiší 728/II, CZ-38925 Vodňany, Czech Republic

**Supplementary Information**

**Table of contents**

| **Sl. No** | **Description** | **Page No.** |
| --- | --- | --- |
| 1 | List of abbrevations | 4 |
| 2 | **Figure S1.** Graphical comparison of the parent pharmaceuticals and selected metabolites measured in the (all) tissues of all plants grown in all soil types after their single and simultaneous application. | 5 |
| 3 | **Table S1.** Basic soil and microbial characteristics: pH_H2O_, pH_CaCl2_, organic carbon content (Cox), cation exchange capacity (CEC), soil hydrolytic acidity (HA), basic cation saturation (BCS), sorption complex saturation (SCS), clay, silt and sand contents. | 6 |
| 4 | **Table S2.** The half-lives (DT_50_; in day) of pharmaceuticals resulted from their single application. | 7 |
| 5 | **Table S3.** Timeline of experiments conducted on the selected plant species. Irrigation with pure water, which is conducted eight times prior to the irrigation with pharmaceuticals, were not included. | 8 |
| 6 | **Table S4**. Dry weights of plant tissues used for the single and simultaneous application of pharmaceuticals. | 9 |
| 7 | **Table S5.** Information about the gradient elution conditions used for the separation of pharmaceuticals. | 11 |
| 8 | **Table S6**. Information about the mass spectrometry conditions used for the analysis of pharmaceuticals. | 12 |
| 9 | **Table S7**. Compounds’ recovery 200 ng per 2 g of soil and 50 ng per 0.05 g of plant tissues (%). | 13 |
| 10 | **Table S8**. Limits of compounds’ quantification (ng g^-1^). | 14 |
| 11 | **Table S9.** Concentration of parent pharmaceuticals and selected metabolites detected in plant parts after their single application. | 15 |
| 12 | **Table S10.** Concentration of parent pharmaceuticals and selected metabolites detected in plant parts after their simultaneous application. | 17 |
| 13 | **Table S11.** Calculated total concentrations of parent pharmaceuticals and selected metabolites in each plants after their single and simultaneous application. | 19 |
| 14 | **Table S12.** Calculated accumulation percentages of pharmaceuticals and their metabolites in plants after their single application. | 21 |
| 15 | **Table S13**. Calculated accumulation percentages of pharmaceuticals and their metabolites in plants after their simultaneous application. | 23 |
| 16 | **Table S14.** Concentration of parent pharmaceuticals and selected metabolites detected in soil after their single application. | 25 |
| 17 | **Table S15.** Concentration of parent pharmaceuticals and selected metabolites detected in soil after their simultaneous application. | 26 |
| 18 | **Table S16.** Bioaccumulation factor (BAF) of pharmaceuticals calculated after their single application. | 27 |
| 19 | **Table S17**. Bioaccumulation factor (BAF) of pharmaceuticals calculated after their simultaneous application. | 29 |

**List of abbreviations**

| CAR | Carbamazepine |
| --- | --- |
| EPC | Carbamazepine 10,11-epoxide |
| TDC | *rac* *trans*-10,11-dihydro-10,11-dihydroxy carbamazepine |
| DHC | 10,11-dihydrocarbamazepine |
| OXC | Oxcarbazepine |
| CIT | Citalopram |
| DCIT | *N-*Desmethylcitalopram |
| CLI | Clindamycin |
| CLIS | Clindamycin Sulfoxide |
| FEX | Fexofenadine |
| IRB | Irbesartan |
| SUL | Sulfamethoxazole |
| N4AS | *N4*-Acetylsulfamethoxazole |
| N1AS | *N1*-Acetylsulfamethoxazole |
| SChS | Stagnic Chernozem Siltic developed on marlite |
| HCh | Haplic Chernozem on loess |
| GP | Greyic Phaeozem on loess |
| HL | Haplic Luvisol on loess |
| HCa | Haplic Cambisol on paragneiss |
| DCa | Dystric Cambisol on orthogneiss |

**
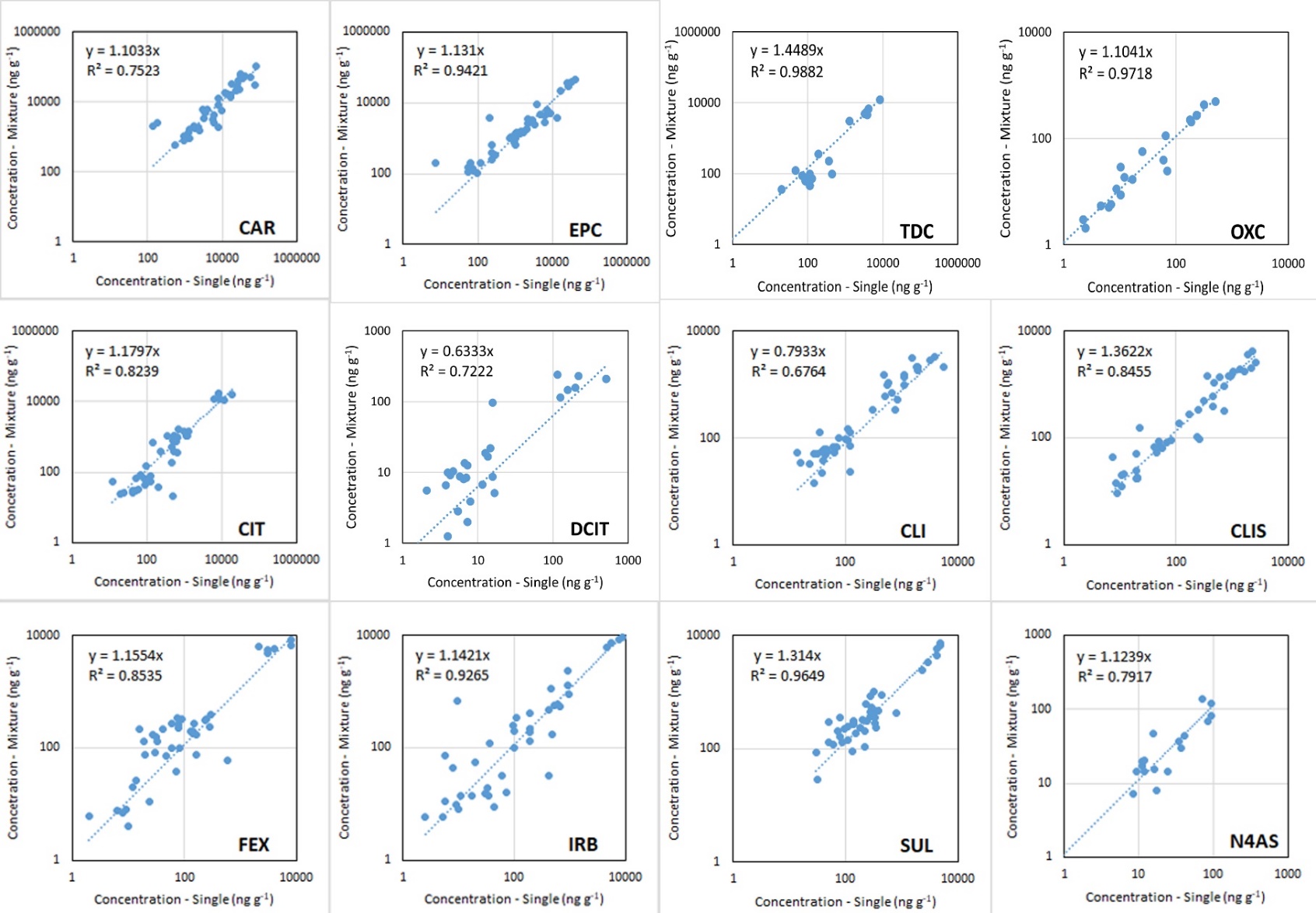
**

**Figure S1**. Graphical comparison of the parent pharmaceuticals and selected metabolites measured in the (all) tissues of all plants grown in all soil types after their single and simultaneous application.

**Table S1**. Basic soil and microbial characteristics: pH_H2O_, pH_CaCl2_, organic carbon content (Cox), cation exchange capacity (CEC), soil hydrolytic acidity (HA), basic cation saturation (BCS), sorption complex saturation (SCS), clay, silt and sand contents (data taken from Kodešová et al. 2020; https://doi.org/10.1016/j.scitotenv.2020.141134).

| **Parameter** | **SChS** | **HCh** | **GP** | **HL** | **HCa** | **DCa** |
| --- | --- | --- | --- | --- | --- | --- |
| **pH_H2O_** | 8.06 | 8.08 | 7.45 | 7.29 | 5.84 | 5.77 |
| **pH_CaCl2_** | 7.41 | 7.35 | 7.14 | 6.29 | 5.36 | 5.26 |
| **Cox** (%) | 2.89 | 1.75 | 1.36 | 1.06 | 1.85 | 2.23 |
| **CEC (**mmol kg^-1^) | 273.0 | 235.0 | 165.0 | 118.0 | 183.0 | 196.0 |
| **HA** (mmol kg^-1^) | 3.61 | 4.21 | 6.61 | 16.8 | 51.7 | 61.3 |
| **BCS** (mmol kg^-1^) | 269.4 | 230.8 | 158.4 | 101.2 | 131.3 | 134.7 |
| **SCS** (%) | 98.7 | 98.2 | 96.0 | 85.7 | 71.8 | 68.7 |
| **Clay** (%) | 20.7 | 36.5 | 17.0 | 12.4 | 18.3 | 19.4 |
| **Silt** (%) | 52.2 | 58.1 | 66.5 | 72.9 | 41.3 | 57.7 |
| **Sand** (%) | 27.2 | 5.4 | 16.5 | 14.7 | 40.4 | 22.9 |

**Table S2**. The half-lives (DT_50_; in day) of pharmaceuticals resulted from their single application (data taken from Kodešová et al. 2020; https://doi.org/10.1016/j.scitotenv.2020.141134).

| **DT_50_ (day)** | **SChS** | **HCh** | **GP** | **HL** | **HCa** | **DCa** |
| --- | --- | --- | --- | --- | --- | --- |
| **CAR** | 140.9 | 100.7 | 101.2 | 93.7 | 99.9 | 91.7 |
| **CIT** | 208.2 | 146.3 | 223.1 | 124.9 | 86.7 | 179.6 |
| **CLI** | 14.7 | 18.4 | 9.1 | 8.6 | 7.4 | 7.4 |
| **FEX** | 9 | 26 | 11.5 | 21.7 | 13.3 | 14.3 |
| **IRB** | 24.2 | 29.2 | 26.4 | 23.1 | 19 | 21.8 |
| **SUL** | 7.8 | 9.6 | 6.8 | 9.2 | 12.7 | 10.8 |

**Table S3**. Timeline of experiments conducted on the selected plant species.

| **Process** | | **Timeline** | | |
| --- | --- | --- | --- | --- |
|  |  | **Onion** | **Spinach** | **Radish** |
| **Sowing** | | April 04, 2017 | April 13, 2017 | April 11, 2017 |
| **Sprouting** | | April 11, 2017 | April 18, 2017 | April 17, 2017 |
| **Irrigation with solutions of different pharmaceuticals (single compound solution) or their mixture** | **Volume (mL)** | | | |
|  | 40 | May 05, 2017 | | |
|  | 30 | May 08, 2017 | | |
|  | 30 | May 11, 2017 | | |
|  | 40 | May 15, 2017 | | |
|  | 30 | May 18, 2017 | | |
|  | 40 | May 22, 2017 | | |
|  | 20 | May 24, 2017 | | |
|  | 30 | May 27, 2017 | | |
|  | 30 | May 30, 2017 | | |
|  | 40 | June 02, 2017 | | |
|  | 20 | June 05, 2017 | | |
|  | 40 | June 08, 2017 | | |
| **Harvesting** | | June 13, 2017 | June 13, 2017 | June 14, 2017 |

**Table S4**. Dry weights of plant tissues used for the single and simultaneous application of pharmaceuticals.

| **Plant part** | **Soil type** | **Dry weight (g)** | | | | | | |
| --- | --- | --- | --- | --- | --- | --- | --- | --- |
|  |  | **Single application** | | | | | | **Simultaneous application** |
|  |  | **CAR** | **CIT** | **CLI** | **FEX** | **IRB** | **SUL** |  |
| **Onion Roots** | SChS | 0.98 | 0.77 | 1.08 | 0.77 | 0.87 | 0.80 | 0.81 |
|  | HCh | 0.95 | 0.81 | 0.88 | 0.72 | 0.74 | 0.86 | 0.77 |
|  | GP | 1.17 | 0.96 | 1.06 | 0.83 | 1.16 | 0.86 | 0.95 |
|  | HL | 0.90 | 0.90 | 1.03 | 0.74 | 0.73 | 0.79 | 0.88 |
|  | HCa | 0.85 | 1.03 | 0.93 | 0.77 | 0.82 | 0.80 | 0.88 |
|  | DCa | 1.27 | 1.04 | 1.00 | 0.78 | 1.15 | 1.00 | 1.07 |
| **Onion Bulb** | SChS | 3.05 | 3.35 | 4.28 | 2.43 | 2.85 | 3.78 | 3.48 |
|  | HCh | 1.75 | 1.76 | 2.18 | 1.39 | 1.83 | 2.80 | 2.84 |
|  | GP | 3.96 | 4.56 | 2.58 | 1.83 | 2.30 | 2.32 | 3.71 |
|  | HL | 2.13 | 2.50 | 2.42 | 1.69 | 2.55 | 2.46 | 2.58 |
|  | HCa | 1.99 | 3.17 | 1.97 | 1.65 | 3.08 | 2.29 | 2.84 |
|  | DCa | 2.88 | 3.20 | 2.16 | 1.33 | 3.03 | 2.74 | 3.72 |
| **Onion Leaves** | SChS | 3.08 | 2.53 | 3.00 | 2.61 | 2.82 | 2.89 | 2.47 |
|  | HCh | 2.31 | 2.12 | 3.02 | 1.93 | 2.00 | 2.24 | 1.88 |
|  | GP | 2.78 | 2.44 | 2.14 | 2.05 | 2.78 | 2.01 | 2.10 |
|  | HL | 2.17 | 2.17 | 2.48 | 1.79 | 2.16 | 2.31 | 2.15 |
|  | HCa | 1.95 | 2.69 | 2.57 | 2.19 | 2.24 | 2.21 | 2.00 |
|  | DCa | 2.44 | 2.66 | 2.27 | 1.66 | 2.14 | 2.33 | 2.09 |
| **Spinach Roots** | SChS | 0.80 | 0.85 | 0.93 | 0.90 | 0.84 | 1.00 | NA |
|  | HCh | 0.98 | 0.87 | 0.88 | 0.84 | 0.83 | 0.86 | 0.83 |
|  | GP | 0.97 | 1.32 | 0.98 | 1.07 | 0.99 | 0.96 | 0.96 |
|  | HL | 1.07 | 1.04 | 0.95 | 0.90 | 0.88 | 0.84 | 0.87 |
|  | HCa | 1.28 | 1.24 | 1.17 | 1.00 | 0.98 | 0.97 | 0.98 |
|  | DCa | 1.37 | 1.55 | 1.23 | 0.95 | 1.22 | 1.04 | 1.14 |
| **Spinach Leaves** | SChS | 1.88 | 1.55 | 2.10 | 2.16 | 1.40 | 1.49 | NA |
|  | HCh | 1.65 | 1.79 | 1.68 | 1.67 | 1.51 | 1.53 | 1.48 |
|  | GP | 1.88 | 2.58 | 1.90 | 2.32 | 2.16 | 2.13 | 1.86 |
|  | HL | 1.84 | 1.89 | 1.75 | 1.88 | 1.90 | 1.71 | 1.68 |
|  | HCa | 2.26 | 2.72 | 2.31 | 2.55 | 2.24 | 2.20 | 2.33 |
|  | DCa | 2.57 | 2.44 | 2.53 | 2.40 | 2.28 | 2.13 | 2.46 |
| **Radish Roots + Bulbs** | SChS | 1.58 | 1.51 | 1.47 | 1.40 | 1.37 | 1.34 | 1.58 |
|  | HCh | 1.36 | 1.34 | 1.27 | 1.68 | 1.49 | 1.25 | 1.58 |
|  | GP | 1.58 | 1.53 | 1.44 | 1.72 | 1.53 | 1.60 | 1.62 |
|  | HL | 0.98 | 1.07 | 1.02 | 1.17 | 1.36 | 1.11 | 1.27 |
|  | HCa | 1.23 | 1.07 | 1.23 | 1.28 | 1.15 | 1.25 | 1.51 |
|  | DCa | 1.40 | 1.29 | 1.28 | 1.51 | 1.50 | 1.68 | 1.76 |
| **Radish Leaves** | SChS | 1.95 | 1.80 | 1.96 | 2.07 | 2.08 | 1.80 | 1.69 |
|  | HCh | 1.72 | 1.45 | 1.67 | 2.04 | 2.01 | 1.70 | 2.08 |
|  | GP | 1.86 | 1.80 | 2.00 | 1.96 | 1.87 | 1.78 | 2.57 |
|  | HL | 1.40 | 1.45 | 1.40 | 1.62 | 2.00 | 1.49 | 1.63 |
|  | HCa | 1.48 | 1.61 | 1.61 | 1.72 | 1.44 | 1.96 | 1.84 |
|  | DCa | 1.65 | 1.67 | 1.93 | 1.68 | 1.63 | 1.69 | 1.43 |

**Table S5**. Information about the gradient elution conditions used for the separation of pharmaceuticals.

| Time (min) | [H_2_O (with 0.1 % formic acid, FA)] (%) | [Acetonitrile (with 0.1 % formic acid, FA)] (%) | Flow (µL min^-1^) |
| --- | --- | --- | --- |
| 0 | 100 | 0 | 350 |
| 1 | 100 | 0 | 350 |
| 4 | 75 | 25 | 350 |
| 8 | 40 | 60 | 450 |
| 10 | 0 | 100 | 450 |
| 11.5 | 0 | 100 | 450 |
| 11.55 | 100 | 0 | 350 |
| 13 | 100 | 0 | 350 |

**Table S6**. Information about the mass spectrometry conditions used for the analysis of pharmaceuticals.

| **Compound** | **Parent ion** | **m/z quan** | **m/z qual** | **Retention time [min]** | |
| --- | --- | --- | --- | --- | --- |
| Carbamazepine | 237.1022 | 194.0964 | 192.0808 | 5.96 |  |
| Carbamazepine 10,11-epoxide | 253.0972 | 210.0913 | 180.0811 | 5.30 |  |
| *rac trans*-10,11-dihydro-10,11-dihydroxy carbamazepine | 271.1177 | 254.0813 | 210.0915 | 4.66 |  |
| 10,11-dihydrocarbamazepine | 239.1179 | 194.0965 | 222.0913 | 6.02 |  |
| Oxcarbazepine | 253.0972 | 236.0707 | 208.0757 | 5.48 |  |
| Citalopram | 325.1711 | 109.0451 | 262.1025 | 6.05 | |
| *N*-Desmethylcitalopram | 311.1554 | 109.0450 | 262.1024 | 5.96 | |
| Clindamycin | 425.1872 | 126.1278 | 377.1838 | 5.20 | |
| Clindamycin Sulfoxide | 441.1821 | 377.1835 | 126.1279 | 4.43 | |
| Fexofenadine | 502.2952 | 466.2742 | 484.2846 | 6.63 | |
| Irbesartan | 429.23974 | 207.0917 | 195.1490 | 6.40 | |
| Sulfamethoxazole | 254.0594 | 156.0112 | 108.0447 | 4.71 | |
| *N1*-Acetylsulfamethoxazole | 296.0699 | 254.0585 | 156.0111 | 5.69 | |
| *N4*-Acetylsulfamethoxazole | 296.0699 | 198.0221 | 134.0600 | 5.05 | |

**Table S7**. Compounds’ recovery 200 ng per 2 g of soil and 50 ng per 0.05 g of plant tissues (%).

| Compound | Onion | | | Spinach | | Radish | | Soil |  |
| --- | --- | --- | --- | --- | --- | --- | --- | --- | --- |
|  | Roots | Bulbs | Leaves | Roots | Leaves | Roots+ Bulbs | Leaves |  |  |
| Carbamazepine | 92% | 92% | 120% | 91% | 95% | 93% | 92% | 105% |  |
| Carbamazepine 10,11-epoxide | 119% | 121% | 106% | 106% | 102% | 105% | 88% | 138% |  |
| *rac trans*-10,11-dihydro-10,11-dihydroxy carbamazepine | 96% | 91% | 99% | 90% | 116% | 101% | 102% | 107% |  |
| 10,11-dihydrocarbamazepine | 81% | 122% | 107% | 111% | 82% | 72% | 67% | 65% |  |
| Oxcarbazepine | 103% | 104% | 99% | 89% | 85% | 86% | 90% | 71% |  |
| Citalopram | 98% | 97% | 100% | 99% | 98% | 99% | 96% | 109% |  |
| *N*-Desmethylcitalopram | 86% | 99% | 103% | 98% | 90% | 91% | 97% | 126% |  |
| Clindamycin | 92% | 90% | 95% | 85% | 92% | 93% | 94% | 91% |  |
| Clindamycin Sulfoxide | 96% | 91% | 123% | 97% | 79% | 81% | 90% | 61% |  |
| Fexofenadine | 99% | 94% | 108% | 89% | 91% | 94% | 94% | 91% |  |
| Irbesartan | 100% | 95% | 102% | 98% | 106% | 98% | 95% | 106% |  |
| Sulfamethoxazole | 104% | 107% | 108% | 101% | 108% | 101% | 105% | 100% |  |
| *N1*-Acetylsulfamethoxazole | 100% | 96% | 144% | 123% | 82% | 131% | 99% | 92% |  |
| *N4*-Acetylsulfamethoxazole | 98% | 107% | 104% | 109% | 103% | 86% | 86% | 81% | |

**Table S8**. Limits of compounds’ quantification (ng g^-1^).

|  | Onion | | | | | | | Spinach | | | | Radish | | | | Soil | |
| --- | --- | --- | --- | --- | --- | --- | --- | --- | --- | --- | --- | --- | --- | --- | --- | --- | --- |
| Compound | Roots | | | Bulbs | | Leaves | | Roots | | Leaves | | Roots + Bulbs | | Leaves | |  |  |
|  | | min | max | min | max | min | max | min | max | min | max | min | max | min | max | min | max |
| Carbamazepine | | 1.1 | 2.9 | 1.1 | 2.4 | 1.4 | 3.2 | 0.33 | 0.63 | 0.5 | 0.95 | 1.2 | 2.2 | 0.35 | 0.87 | 0.13 | 0.47 |
| Carbamazepine 10,11-epoxide | | 5.8 | 16 | 6 | 13 | 4.9 | 11 | 3.7 | 7 | 3.1 | 5.9 | 4.1 | 7.7 | 1.6 | 3.8 | 0.069 | 0.52 |
| *rac trans*-10,11-dihydro-10,11-dihydroxy carbamazepine | | 0.43 | 1.2 | 0.46 | 1 | 0.99 | 2.3 | 0.61 | 1.2 | 0.35 | 0.67 | 0.95 | 1.8 | 0.61 | 1.5 | 0.14 | 1.4 |
| 10,11-dihydrocarbamazepine | | 33 | 89 | 87 | 190 | 51 | 120 | 38 | 73 | 17 | 44 | 38 | 71 | 18 | 43 | 0.12 | 0.8 |
| Oxcarbazepine | | 1.1 | 2.9 | 5.9 | 13 | 5.7 | 13 | 6.8 | 13 | 8.3 | 16 | 4 | 7.6 | 3.5 | 8.5 | 0.14 | 0.62 |
| Citalopram | | 0.69 | 1.3 | 0.82 | 1.6 | 0.77 | 1.3 | 0.39 | 0.82 | 0.62 | 1 | 0.29 | 0.56 | 0.68 | 1.3 | 0.14 | 1.7 |
| *N*-Desmethylcitalopram | | 0.2 | 0.54 | 0.17 | 0.58 | 0.2 | 0.52 | 0.28 | 0.69 | 0.2 | 0.52 | 0.15 | 0.41 | 0.21 | 0.5 | 0.36 | 3.6 |
| Clindamycin | | 1.6 | 4.3 | 1.5 | 5.2 | 1.6 | 5.8 | 2.4 | 5.9 | 2.1 | 5.3 | 1.2 | 3.2 | 1.6 | 3.8 | 0.17 | 1.7 |
| Clindamycin Sulfoxide | | 0.64 | 1.2 | 0.81 | 1.6 | 0.35 | 0.56 | 0.79 | 1.7 | 0.68 | 1.1 | 0.72 | 1.4 | 0.19 | 0.36 | 0.16 | 1.5 |
| Fexofenadine | | 0.34 | 0.72 | 1.8 | 4.5 | 1.8 | 4.6 | 0.26 | 0.54 | 2.2 | 4 | 1.6 | 4.4 | 1.3 | 3.7 | 0.081 | 0.65 |
| Irbesartan | | 0.32 | 0.56 | 0.68 | 1.4 | 0.34 | 0.57 | 0.39 | 0.61 | 0.36 | 0.58 | 0.38 | 0.72 | 0.74 | 1.4 | 0.085 | 0.39 |
| Sulfamethoxazole | | 5.2 | 12 | 4.4 | 7.7 | 5.1 | 9.7 | 5.8 | 11 | 3.7 | 7.2 | 8 | 19 | 7.7 | 20 | 0.19 | 0.9 |
| *N1*-Acetylsulfamethoxazole | | 180 | 390 | 130 | 230 | 9.7 | 18 | 4 | 7.7 | 6.7 | 13 | 110 | 260 | 11 | 28 | 1.1 | 12 |
| *N4*-Acetylsulfamethoxazole | | 7.1 | 16 | 6.1 | 11 | 14 | 28 | 7.1 | 13 | 4.7 | 9.2 | 4.1 | 9.7 | 7.7 | 20 | 0.22 | 1 |

**Table S9**. Concentration of parent pharmaceuticals and selected metabolites detected in plant parts after their single application.

| **Plant parts** | **Soil type** | **Concentration (ng g^-1^)** | | | | | | | | | | | | | |
| --- | --- | --- | --- | --- | --- | --- | --- | --- | --- | --- | --- | --- | --- | --- | --- |
|  |  | **CAR** | **EPC** | **TDC** | **OXC** | **DHC** | **CIT** | **DCIT** | **CLI** | **CLIS** | **FEX** | **IRB** | **SUL** | **N4AS** | **N1AS** |
| **Onion Roots** | **SChS** | 17000 | 1405 | <LOQ | <LOQ | <LOQ | 8300 | <LOQ | 1800 | 1349 | 3000 | 8900 | 2300 | 35 | <LOQ |
|  | **HCh** | 13000 | 777 | <LOQ | <LOQ | <LOQ | 6400 | <LOQ | 5400 | 1638 | 2100 | 6900 | 2900 | 15 | <LOQ |
|  | **GP** | 24000 | 2060 | <LOQ | <LOQ | <LOQ | 12000 | <LOQ | 3100 | 2216 | 3000 | 11000 | 4200 | 41 | <LOQ |
|  | **HL** | 23000 | 1311 | <LOQ | <LOQ | <LOQ | 9100 | <LOQ | 3900 | 2602 | 7900 | 7700 | 4800 | 86 | <LOQ |
|  | **HCa** | 16000 | 1030 | <LOQ | <LOQ | <LOQ | 8400 | <LOQ | 1500 | 1927 | 7900 | 5600 | 4200 | 94 | <LOQ |
|  | **DCa** | 17000 | 1124 | <LOQ | <LOQ | <LOQ | 19000 | <LOQ | 1900 | 2313 | 4100 | 4600 | 4900 | 70 | <LOQ |
| **Onion Bulb** | **SChS** | 540 | 702 | <LOQ | <LOQ | <LOQ | 20 | <LOQ | 39 | 9 | 2 | 99 | 150 | 0 | <LOQ |
|  | **HCh** | 940 | 937 | <LOQ | <LOQ | <LOQ | 41 | <LOQ | 44 | 11 | 9 | 94 | 180 | 9 | <LOQ |
|  | **GP** | 1300 | 1405 | <LOQ | <LOQ | <LOQ | 210 | <LOQ | 42 | 12 | 7 | 190 | 270 | 17 | <LOQ |
|  | **HL** | 2300 | 1686 | <LOQ | <LOQ | <LOQ | 52 | <LOQ | 64 | 19 | 24 | 99 | 330 | <LOQ | <LOQ |
|  | **HCa** | 1200 | 1030 | <LOQ | <LOQ | <LOQ | 25 | <LOQ | 32 | 20 | 8 | 5 | 200 | <LOQ | <LOQ |
|  | **DCa** | 1300 | 1030 | <LOQ | <LOQ | <LOQ | 42 | <LOQ | 16 | 11 | 10 | 3 | 140 | <LOQ | <LOQ |
| **Onion Leaves** | **SChS** | 12000 | 15922 | 1311 | 66 | <LOQ | 620 | 4 | 570 | 472 | 28 | 33 | 140 | <LOQ | <LOQ |
|  | **HCh** | 27000 | 26224 | 3846 | 187 | <LOQ | 1200 | 6 | 1100 | 964 | 16 | 20 | 280 | <LOQ | <LOQ |
|  | **GP** | 30000 | 33717 | 4196 | 318 | <LOQ | 650 | 5 | 1100 | 858 | 30 | 9 | 290 | <LOQ | <LOQ |
|  | **HL** | 80000 | 41209 | 8479 | 506 | <LOQ | 1300 | 7 | 1900 | 1060 | 130 | 6 | 430 | <LOQ | <LOQ |
|  | **HCa** | 34000 | 26224 | 3322 | 178 | <LOQ | 520 | 4 | 470 | 357 | 150 | 8 | 320 | <LOQ | <LOQ |
|  | **DCa** | 39000 | 25288 | 3759 | 234 | <LOQ | 1100 | 7 | 540 | 597 | 60 | 11 | 230 | <LOQ | <LOQ |
| **Spinach Roots** | **SChS** | 140 | 7 | <LOQ | <LOQ | <LOQ | 67 | <LOQ | 120 | 80 | 79 | 190 | 110 | 8 | <LOQ |
|  | **HCh** | 920 | 55 | <LOQ | <LOQ | <LOQ | 52 | 2 | 38 | 19 | 82 | 520 | 270 | 12 | <LOQ |
|  | **GP** | 3200 | 234 | <LOQ | <LOQ | <LOQ | 510 | 13 | 28 | 20 | 73 | 470 | 380 | 12 | <LOQ |
|  | **HL** | 5500 | 290 | <LOQ | <LOQ | <LOQ | 520 | 5 | 38 | 51 | 240 | 930 | 780 | 38 | <LOQ |
|  | **HCa** | 1700 | 64 | <LOQ | <LOQ | <LOQ | 86 | <LOQ | 46 | 49 | 140 | 190 | 51 | <LOQ | <LOQ |
|  | **DCa** | 1900 | 70 | <LOQ | <LOQ | <LOQ | 580 | 11 | 110 | 241 | 280 | 110 | 73 | <LOQ | <LOQ |
| **Spinach Leaves** | **SChS** | 180 | 206 | <LOQ | <LOQ | <LOQ | 12 | <LOQ | 14 | 22 | 61 | 34 | 49 | 8 | <LOQ |
|  | **HCh** | 2400 | 2248 | 20 | <LOQ | <LOQ | 130 | 4 | 60 | 67 | 160 | 420 | 96 | 11 | <LOQ |
|  | **GP** | 9800 | 8897 | 87 | <LOQ | <LOQ | 61 | 2 | 23 | 57 | 160 | 17 | 85 | <LOQ | <LOQ |
|  | **HL** | 5800 | 4870 | 105 | <LOQ | <LOQ | 130 | 8 | 69 | 173 | 300 | 61 | 240 | <LOQ | <LOQ |
|  | **HCa** | 5900 | 6275 | 114 | <LOQ | <LOQ | 95 | 7 | 47 | 116 | 250 | 30 | 79 | <LOQ | <LOQ |
|  | **DCa** | 7800 | 6181 | 114 | <LOQ | <LOQ | 88 | 2 | 120 | 260 | 600 | 10 | 30 | <LOQ | <LOQ |
| **Radish Roots + Bulbs** | **SChS** | 3200 | 94 | <LOQ | <LOQ | <LOQ | 360 | 2 | 74 | 19 | 33 | 680 | 220 | <LOQ | <LOQ |
|  | **HCh** | 3000 | 53 | <LOQ | <LOQ | <LOQ | 610 | 4 | 110 | 41 | 140 | 920 | 300 | <LOQ | <LOQ |
|  | **GP** | 7600 | 244 | <LOQ | <LOQ | <LOQ | 690 | 6 | 120 | 45 | 41 | 980 | 330 | <LOQ | <LOQ |
|  | **HL** | 7600 | 234 | <LOQ | <LOQ | <LOQ | 140 | <LOQ | 35 | 7 | 78 | 410 | 350 | <LOQ | <LOQ |
|  | **HCa** | 3400 | 76 | <LOQ | <LOQ | <LOQ | 94 | <LOQ | 28 | 9 | 47 | 190 | 220 | <LOQ | <LOQ |
|  | **DCa** | 3800 | 122 | <LOQ | <LOQ | <LOQ | 990 | 13 | 99 | 51 | 76 | 600 | 330 | <LOQ | <LOQ |
| **Radish Leaves** | **SChS** | 25000 | 2903 | 71 | 12 | <LOQ | 450 | 14 | 510 | 318 | 19 | 37 | 130 | <LOQ | <LOQ |
|  | **HCh** | 18000 | 2154 | 47 | 10 | <LOQ | 540 | 16 | 1100 | 443 | 89 | 9 | 140 | <LOQ | <LOQ |
|  | **GP** | 73000 | 13112 | 446 | 70 | <LOQ | 660 | 17 | 770 | 723 | 12 | 43 | 32 | <LOQ | <LOQ |
|  | **HL** | 56000 | 7399 | 367 | 60 | <LOQ | 460 | 16 | 830 | 453 | 20 | 6 | 81 | <LOQ | <LOQ |
|  | **HCa** | 29000 | 3278 | 131 | 17 | <LOQ | 230 | 7 | 300 | 251 | 32 | 490 | 59 | <LOQ | <LOQ |
|  | **DCa** | 30000 | 3934 | 192 | 25 | <LOQ | 510 | 15 | 660 | 713 | 14 | 74 | 110 | <LOQ | <LOQ |

**LOQ**: Limit of Quantification

**Table S10**. Concentration of parent pharmaceuticals and selected metabolites detected in plant parts after their simultaneous application.

| **Plant parts** | **Soil type** | **Concentration (ng g^-1^)** | | | | | | | | | | | | | |
| --- | --- | --- | --- | --- | --- | --- | --- | --- | --- | --- | --- | --- | --- | --- | --- |
|  |  | **CAR** | **EPC** | **TDC** | **OXC** | **DHC** | **CIT** | **DCIT** | **CLI** | **CLIS** | **FEX** | **IRB** | **SUL** | **N4AS** | **N1AS** |
| **Onion Roots** | **SChS** | 14000 | 1592 | <LOQ | 3 | 17000 | 240 | 2100 | 1927 | 4800 | 9400 | 2400 | 37 | <LOQ | <LOQ |
|  | **HCh** | 17000 | 1124 | <LOQ | <LOQ | 12000 | 230 | 2200 | 1735 | 6300 | 12000 | 3400 | 47 | <LOQ | <LOQ |
|  | **GP** | 22000 | 1779 | <LOQ | 6 | 11000 | 157 | 2900 | 2024 | 5700 | 9800 | 4300 | 44 | <LOQ | <LOQ |
|  | **HL** | 21000 | 1311 | <LOQ | 5 | 12000 | 115 | 3400 | 2602 | 8600 | 8600 | 6600 | 69 | <LOQ | <LOQ |
|  | **HCa** | 16000 | 1311 | <LOQ | 5 | 17000 | 146 | 3100 | 3662 | 6700 | 7300 | 5900 | 120 | <LOQ | <LOQ |
|  | **DCa** | 16000 | 1499 | <LOQ | 2 | 16000 | 209 | 2200 | 4240 | 5900 | 6200 | 7400 | 137 | <LOQ | <LOQ |
| **Onion Bulb** | **SChS** | 600 | 1030 | <LOQ | <LOQ | 25 | <LOQ | 37 | 14 | 6 | 100 | 190 | <LOQ | <LOQ | <LOQ |
|  | **HCh** | 780 | 777 | <LOQ | <LOQ | 26 | <LOQ | 49 | 13 | 8 | 250 | 240 | <LOQ | <LOQ | <LOQ |
|  | **GP** | 1700 | 1499 | <LOQ | 11 | 38 | <LOQ | 61 | 21 | 8 | 410 | 370 | 8 | <LOQ | <LOQ |
|  | **HL** | 1900 | 1405 | <LOQ | 9 | 31 | <LOQ | 53 | 17 | 11 | 200 | 460 | <LOQ | <LOQ | <LOQ |
|  | **HCa** | 1300 | 1030 | <LOQ | <LOQ | 26 | <LOQ | 50 | 17 | 7 | 6 | 320 | <LOQ | <LOQ | <LOQ |
|  | **DCa** | 900 | 656 | <LOQ | <LOQ | 30 | <LOQ | 34 | 20 | 4 | 6 | 270 | <LOQ | <LOQ | <LOQ |
| **Onion Leaves** | **SChS** | 18000 | 22478 | 3060 | 112 | 870 | 9 | 1100 | 1060 | 170 | 19 | 310 | <LOQ | <LOQ | <LOQ |
|  | **HCh** | 40000 | 29034 | 4546 | 206 | 1100 | 9 | 1500 | 1446 | 220 | 56 | 440 | <LOQ | <LOQ | <LOQ |
|  | **GP** | 63000 | 38400 | 6731 | 431 | 1000 | 10 | 1400 | 1446 | 82 | 10 | 540 | <LOQ | <LOQ | <LOQ |
|  | **HL** | 110000 | 44019 | 12238 | 496 | 1400 | 13 | 1900 | 1735 | 200 | 11 | 900 | <LOQ | <LOQ | <LOQ |
|  | **HCa** | 48000 | 31844 | 4895 | 225 | 1100 | 10 | 1500 | 1446 | 270 | 43 | 1000 | <LOQ | <LOQ | <LOQ |
|  | **DCa** | 54000 | 37463 | 5507 | 272 | 1100 | 8 | 1000 | 1349 | 100 | 14 | 600 | <LOQ | <LOQ | <LOQ |
| **Spinach Roots** | **SChS** | 2000 | 206 | <LOQ | <LOQ | 81 | <LOQ | 72 | 92 | 230 | 220 | 140 | 0 | <LOQ | <LOQ |
|  | **HCh** | 1100 | 112 | <LOQ | <LOQ | 67 | <LOQ | 22 | 24 | 100 | 560 | 830 | 15 | <LOQ | <LOQ |
|  | **GP** | 3400 | 646 | <LOQ | <LOQ | 21 | <LOQ | 14 | 18 | 38 | 1100 | 460 | 21 | <LOQ | <LOQ |
|  | **HL** | 3300 | 337 | <LOQ | <LOQ | 400 | 3 | 57 | 70 | 310 | 1300 | 430 | 30 | <LOQ | <LOQ |
|  | **HCa** | 2000 | 197 | <LOQ | <LOQ | 69 | 1 | 62 | 88 | 200 | 190 | 130 | 0 | <LOQ | <LOQ |
|  | **DCa** | 1800 | 140 | <LOQ | <LOQ | 760 | 7 | 92 | 106 | 240 | 340 | 200 | 17 | <LOQ | <LOQ |
| **Spinach Leaves** | **SChS** | 2500 | 3653 | 70 | <LOQ | 55 | 2 | 52 | 154 | 270 | 14 | 300 | 7 | <LOQ | <LOQ |
|  | **HCh** | 1500 | 2622 | 36 | <LOQ | 54 | 1 | 68 | 84 | 170 | 32 | 220 | 20 | <LOQ | <LOQ |
|  | **GP** | 5600 | 4870 | 62 | <LOQ | 33 | 0 | 33 | 66 | 76 | 14 | 130 | 9 | <LOQ | <LOQ |
|  | **HL** | 4400 | 4589 | 71 | <LOQ | 77 | 4 | 69 | 270 | 390 | 31 | 310 | 82 | <LOQ | <LOQ |
|  | **HCa** | 2600 | 4308 | 96 | <LOQ | 64 | 2 | 57 | 183 | 330 | 15 | 360 | 15 | <LOQ | <LOQ |
|  | **DCa** | 1900 | 2716 | 45 | <LOQ | 42 | <LOQ | 23 | 96 | 60 | 8 | 87 | 9 | <LOQ | <LOQ |
| **Radish Roots + Bulbs** | **SChS** | 5700 | 103 | <LOQ | <LOQ | 1100 | 6 | 100 | 50 | 130 | 540 | 200 | <LOQ | <LOQ | <LOQ |
|  | **HCh** | 6000 | 150 | <LOQ | <LOQ | 730 | 7 | 150 | 68 | 180 | 2300 | 380 | 22 | <LOQ | <LOQ |
|  | **GP** | 13000 | 384 | <LOQ | <LOQ | 1700 | 8 | 130 | 53 | 220 | 900 | 280 | <LOQ | <LOQ | <LOQ |
|  | **HL** | 8100 | 253 | <LOQ | <LOQ | 690 | 5 | 130 | 43 | 260 | 470 | 240 | 15 | <LOQ | <LOQ |
|  | **HCa** | 4800 | 122 | <LOQ | <LOQ | 150 | <LOQ | 50 | 9 | 71 | 130 | 110 | <LOQ | <LOQ | <LOQ |
|  | **DCa** | 6200 | 206 | <LOQ | <LOQ | 1400 | 19 | 94 | 67 | 340 | 600 | 350 | 15 | <LOQ | <LOQ |
| **Radish Leaves** | **SChS** | 28000 | 3278 | 87 | 19 | 510 | 17 | 600 | 491 | 130 | 120 | 89 | <LOQ | <LOQ | <LOQ |
|  | **HCh** | 34000 | 3465 | 122 | 29 | 1100 | 97 | 1000 | 607 | 320 | 680 | 300 | 14 | <LOQ | <LOQ |
|  | **GP** | 31000 | 3840 | 96 | 24 | 370 | 5 | 340 | 318 | 20 | 9 | 29 | <LOQ | <LOQ | <LOQ |
|  | **HL** | 53000 | 6275 | 227 | 39 | 190 | 9 | 520 | 385 | 75 | 71 | 160 | 17 | <LOQ | <LOQ |
|  | **HCa** | 23000 | 2435 | 72 | 17 | 390 | 14 | 340 | 337 | 160 | 170 | 120 | <LOQ | <LOQ | <LOQ |
|  | **DCa** | 59000 | 9366 | 358 | 57 | 820 | 22 | 690 | 944 | 26 | 16 | 250 | 21 | <LOQ | <LOQ |

**LOQ**: Limit of Quantification

**Table S11**. Calculated total concentrations of parent pharmaceuticals and selected metabolites in each plants after their single and simultaneous application.

| **Plant** | **Soil type** | **Concentration (ng g^-1^)** | | | | | | | | | | | | | |
| --- | --- | --- | --- | --- | --- | --- | --- | --- | --- | --- | --- | --- | --- | --- | --- |
|  |  | **CAR** | **EPC** | **TDC** | **OXC** | **DHC** | **CIT** | **DCIT** | **CLI** | **CLIS** | **FEX** | **IRB** | **SUL** | **N4AS** | **N1AS** |
| **Single application** | | | | | | | | | | | | | | | |
| **Onion** | SChS | 7773 | 7893 | 650 | 31 | 0 | 1207 | 14 | 457 | 361 | 593 | 1241 | 376 | 4 | 0 |
|  | HCh | 15243 | 13417 | 2029 | 92 | 0 | 1663 | 39 | 1344 | 747 | 546 | 1164 | 614 | 8 | 0 |
|  | GP | 14744 | 13729 | 1742 | 125 | 0 | 1767 | 26 | 995 | 757 | 747 | 2119 | 929 | 17 | 0 |
|  | HL | 38308 | 19341 | 4048 | 231 | 0 | 2000 | 22 | 1498 | 937 | 1906 | 1082 | 1007 | 14 | 0 |
|  | HCa | 17179 | 12051 | 1547 | 79 | 0 | 1470 | 24 | 487 | 521 | 1325 | 753 | 854 | 17 | 0 |
|  | DCa | 18284 | 10709 | 1592 | 93 | 0 | 3307 | 76 | 582 | 706 | 946 | 842 | 959 | 14 | 0 |
| **Spinach** | SChS | 168 | 157 | 0 | 0 | 0 | 31 | 0 | 47 | 41 | 66 | 93 | 73 | 10 | 0 |
|  | HCh | 1849 | 1528 | 14 | 0 | 0 | 104 | 3 | 52 | 53 | 134 | 455 | 159 | 13 | 0 |
|  | GP | 7554 | 6352 | 66 | 0 | 0 | 213 | 5 | 25 | 46 | 133 | 159 | 177 | 4 | 0 |
|  | HL | 5690 | 3402 | 76 | 0 | 0 | 268 | 7 | 58 | 135 | 281 | 336 | 418 | 88 | 0 |
|  | HCa | 4381 | 4302 | 83 | 0 | 0 | 92 | 5 | 47 | 97 | 219 | 79 | 70 | 8 | 0 |
|  | DCa | 5748 | 4331 | 85 | 0 | 0 | 279 | 6 | 117 | 263 | 509 | 45 | 44 | 0 | 0 |
| **Radish** | SChS | 15242 | 1757 | 45 | 7 | 0 | 409 | 8 | 323 | 197 | 25 | 292 | 168 | 0 | 0 |
|  | HCh | 11377 | 1310 | 30 | 6 | 0 | 574 | 10 | 672 | 280 | 112 | 397 | 208 | 0 | 0 |
|  | GP | 42962 | 7689 | 276 | 41 | 0 | 674 | 11 | 498 | 456 | 26 | 465 | 173 | 0 | 0 |
|  | HL | 36071 | 4750 | 247 | 38 | 0 | 324 | 9 | 495 | 275 | 44 | 169 | 196 | 20 | 0 |
|  | HCa | 17381 | 1948 | 82 | 10 | 0 | 176 | 4 | 182 | 151 | 38 | 357 | 122 | 4 | 0 |
|  | DCa | 17974 | 2332 | 119 | 15 | 0 | 719 | 13 | 436 | 466 | 43 | 326 | 220 | 9 | 0 |
| **Simultaneous application** | | | | | | | | | | | | | | | |
| **Onion** | SChS | 8563 | 9539 | 1279 | 44 | 0 | 2368 | 31 | 673 | 649 | 640 | 1185 | 499 | 5 | 0 |
|  | HCh | 16485 | 11213 | 1781 | 75 | 0 | 2073 | 34 | 848 | 773 | 963 | 1832 | 752 | 8 | 0 |
|  | GP | 23596 | 13882 | 2392 | 150 | 0 | 1877 | 24 | 876 | 773 | 831 | 1605 | 975 | 12 | 0 |
|  | HL | 46325 | 18922 | 5365 | 208 | 0 | 2433 | 22 | 1286 | 1122 | 1431 | 1445 | 1592 | 13 | 0 |
|  | HCa | 19890 | 12650 | 1958 | 85 | 0 | 3013 | 25 | 1026 | 1118 | 1129 | 1141 | 1416 | 22 | 0 |
|  | DCa | 19379 | 12778 | 1914 | 88 | 0 | 2839 | 34 | 664 | 1121 | 950 | 972 | 1479 | 25 | 0 |
| **Spinach** | SChS | NA | NA | NA | NA | NA | NA | NA | NA | NA | NA | NA | NA | NA | NA |
|  | HCh | 1356 | 1837 | 26 | 0 | 0 | 59 | 1 | 51 | 65 | 145 | 222 | 439 | 21 | 0 |
|  | GP | 4851 | 3665 | 47 | 0 | 0 | 29 | 0 | 27 | 51 | 63 | 384 | 242 | 15 | 0 |
|  | HL | 4025 | 3351 | 53 | 0 | 0 | 187 | 3 | 65 | 209 | 363 | 464 | 351 | 75 | 0 |
|  | HCa | 2422 | 3300 | 77 | 0 | 0 | 65 | 2 | 58 | 161 | 292 | 67 | 292 | 12 | 0 |
|  | DCa | 1868 | 2029 | 36 | 0 | 0 | 269 | 2 | 45 | 103 | 117 | 113 | 123 | 14 | 0 |
| **Radish** | SChS | 17225 | 1862 | 52 | 10 | 0 | 795 | 11 | 358 | 289 | 130 | 323 | 143 | 0 | 0 |
|  | HCh | 21913 | 2172 | 80 | 18 | 0 | 940 | 56 | 633 | 389 | 260 | 1379 | 335 | 20 | 0 |
|  | GP | 24041 | 2673 | 67 | 16 | 0 | 884 | 6 | 259 | 224 | 97 | 353 | 126 | 0 | 0 |
|  | HL | 33337 | 3884 | 146 | 24 | 0 | 409 | 7 | 349 | 245 | 156 | 246 | 195 | 19 | 0 |
|  | HCa | 14796 | 1487 | 45 | 10 | 0 | 282 | 7 | 209 | 197 | 120 | 152 | 115 | 0 | 0 |
|  | DCa | 29869 | 4604 | 184 | 27 | 0 | 1277 | 23 | 608 | 829 | 212 | 344 | 313 | 21 | 0 |

**Table S12**. Calculated accumulation percentages of pharmaceuticals and their metabolites in plants after their single application.

| **Plant part** | **Soil type** | **Accumulation (%)** | | | | | | | | | | | | | |
| --- | --- | --- | --- | --- | --- | --- | --- | --- | --- | --- | --- | --- | --- | --- | --- |
|  |  | **CAR** | **EPC** | **TDC** | **OXC** | **DHC** | **CIT** | **DCIT** | **CLI** | **CLIS** | **FEX** | **IRB** | **SUL** | **N4AS** | **N1AS** |
| **Onion Roots** | **SChS** | 30.1 | 2.6 | 0.0 | 1.1 | NA | 79.6 | 89.1 | 50.9 | 50.1 | 96.7 | 95.4 | 65.4 | 100.0 | NA |
|  | **HCh** | 16.2 | 1.2 | 0.0 | 0.0 | NA | 66.5 | 93.6 | 58.2 | 33.0 | 97.2 | 96.0 | 68.8 | 33.4 | NA |
|  | **GP** | 24.1 | 2.4 | 0.0 | 0.9 | NA | 81.9 | 86.8 | 57.2 | 55.7 | 97.1 | 96.5 | 74.9 | 47.1 | NA |
|  | **HL** | 10.4 | 1.3 | 0.0 | 0.4 | NA | 73.5 | 87.7 | 45.2 | 50.0 | 95.5 | 95.5 | 67.7 | 100.0 | NA |
|  | **Hca** | 16.5 | 1.6 | 0.0 | 1.5 | NA | 85.4 | 93.8 | 52.3 | 65.2 | 94.7 | 99.3 | 74.3 | 100.0 | NA |
|  | **Dca** | 17.9 | 2.2 | 0.0 | 0.5 | NA | 86.6 | 96.6 | 60.1 | 62.6 | 96.6 | 99.4 | 84.2 | 100.0 | NA |
| **Onion Bulb** | **SChS** | 3.0 | 4.1 | 0.0 | 0.0 | NA | 0.8 | 0.0 | 4.4 | 1.3 | 0.2 | 3.5 | 20.2 | 0.0 | NA |
|  | **HCh** | 2.2 | 2.6 | 0.0 | 0.0 | NA | 0.9 | 0.0 | 1.2 | 0.5 | 0.8 | 3.2 | 13.9 | 66.6 | NA |
|  | **GP** | 4.4 | 5.5 | 3.2 | 3.7 | NA | 6.8 | 8.0 | 1.9 | 0.7 | 0.5 | 3.3 | 13.0 | 52.9 | NA |
|  | **HL** | 2.5 | 3.8 | 0.0 | 2.0 | NA | 1.2 | 0.0 | 1.7 | 0.9 | 0.7 | 4.3 | 14.5 | 0.0 | NA |
|  | **Hca** | 2.9 | 3.8 | 0.0 | 0.0 | NA | 0.8 | 0.0 | 2.4 | 1.5 | 0.2 | 0.3 | 10.1 | 0.0 | NA |
|  | **Dca** | 3.1 | 4.5 | 0.0 | 0.0 | NA | 0.6 | 0.0 | 1.1 | 0.6 | 0.4 | 0.1 | 6.6 | 0.0 | NA |
| **Onion Leaves** | **SChS** | 66.9 | 93.3 | 100.0 | 98.9 | NA | 19.5 | 10.9 | 44.8 | 48.7 | 3.1 | 1.1 | 14.4 | 0.0 | NA |
|  | **HCh** | 81.7 | 96.2 | 100.0 | 100.0 | NA | 32.6 | 6.4 | 40.7 | 66.5 | 2.0 | 0.8 | 17.3 | 0.0 | NA |
|  | **GP** | 71.5 | 92.2 | 96.8 | 95.4 | NA | 11.3 | 5.2 | 41.0 | 43.5 | 2.4 | 0.2 | 12.1 | 0.0 | NA |
|  | **HL** | 87.1 | 94.9 | 100.0 | 97.7 | NA | 25.3 | 12.3 | 53.0 | 49.1 | 3.8 | 0.2 | 17.7 | 0.0 | NA |
|  | **Hca** | 80.6 | 94.6 | 100.0 | 98.5 | NA | 13.8 | 6.2 | 45.3 | 33.3 | 5.1 | 0.4 | 15.6 | 0.0 | NA |
|  | **Dca** | 79.0 | 93.4 | 100.0 | 99.5 | NA | 12.8 | 3.4 | 38.8 | 36.7 | 3.0 | 0.4 | 9.2 | 0.0 | NA |
| **Spinach Roots** | **SChS** | 24.9 | 1.5 | NA | NA | NA | 75.4 | NA | 79.1 | 61.5 | 35.0 | 77.0 | 60.1 | 38.4 | NA |
|  | **HCh** | 18.5 | 1.4 | 0.0 | NA | NA | 16.3 | 22.0 | 24.9 | 13.0 | 20.5 | 40.5 | 61.3 | 37.7 | NA |
|  | **GP** | 14.4 | 1.3 | 0.0 | NA | NA | 81.1 | 79.3 | 38.6 | 15.5 | 17.4 | 92.7 | 66.8 | 100.0 | NA |
|  | **HL** | 35.5 | 3.4 | 0.0 | NA | NA | 68.8 | 27.4 | 23.0 | 13.8 | 27.7 | 87.6 | 61.5 | 16.4 | NA |
|  | **Hca** | 14.0 | 0.6 | 0.0 | NA | NA | 29.2 | 0.0 | 33.1 | 17.7 | 18.0 | 73.5 | 22.2 | 0.0 | NA |
|  | **Dca** | 11.5 | 0.6 | 0.0 | NA | NA | 80.7 | 76.9 | 30.8 | 31.0 | 15.6 | 85.6 | 54.3 | NA | NA |
| **Spinach Leaves** | **SChS** | 75.1 | 98.5 | NA | NA | NA | 24.6 | NA | 20.9 | 38.5 | 65.0 | 23.0 | 39.9 | 61.6 | NA |
|  | **HCh** | 81.5 | 98.6 | 100.0 | NA | NA | 83.7 | 78.0 | 75.1 | 87.0 | 79.5 | 59.5 | 38.7 | 62.3 | NA |
|  | **GP** | 85.6 | 98.7 | 100.0 | NA | NA | 18.9 | 20.7 | 61.4 | 84.5 | 82.6 | 7.3 | 33.2 | 0.0 | NA |
|  | **HL** | 64.5 | 96.6 | 100.0 | NA | NA | 31.2 | 72.6 | 77.0 | 86.2 | 72.3 | 12.4 | 38.5 | 83.6 | NA |
|  | **Hca** | 86.0 | 99.4 | 100.0 | NA | NA | 70.8 | 100.0 | 66.9 | 82.3 | 82.0 | 26.5 | 77.8 | 100.0 | NA |
|  | **Dca** | 88.5 | 99.4 | 100.0 | NA | NA | 19.3 | 23.1 | 69.2 | 69.0 | 84.4 | 14.4 | 45.7 | NA | NA |
| **Radish Roots + Bulbs** | **SChS** | 9.4 | 2.5 | 0.0 | 0.0 | NA | 40.2 | 11.4 | 9.8 | 4.3 | 54.0 | 92.4 | 55.7 | NA | NA |
|  | **HCh** | 11.6 | 1.9 | 0.0 | 0.0 | NA | 51.1 | 18.2 | 7.1 | 6.6 | 56.4 | 98.7 | 61.2 | NA | NA |
|  | **GP** | 8.1 | 1.6 | 0.0 | 0.0 | NA | 47.1 | 24.8 | 10.1 | 4.3 | 75.0 | 94.9 | 90.3 | NA | NA |
|  | **HL** | 8.7 | 2.2 | 0.0 | 0.0 | NA | 18.3 | 0.0 | 3.0 | 1.2 | 73.8 | 98.0 | 76.3 | 62.5 | NA |
|  | **Hca** | 8.9 | 1.9 | 0.0 | 0.0 | NA | 21.4 | 0.0 | 6.7 | 2.6 | 52.2 | 23.6 | 70.4 | 100.0 | NA |
|  | **Dca** | 9.7 | 2.6 | 0.0 | 0.0 | NA | 60.0 | 39.8 | 9.0 | 4.5 | 83.0 | 88.2 | 74.9 | 100.0 | NA |
| **Radish Leaves** | **SChS** | 90.6 | 97.5 | 100.0 | 100.0 | NA | 59.8 | 88.6 | 90.2 | 95.7 | 46.0 | 7.6 | 44.3 | NA | NA |
|  | **HCh** | 88.4 | 98.1 | 100.0 | 100.0 | NA | 48.9 | 81.8 | 92.9 | 93.4 | 43.6 | 1.3 | 38.8 | NA | NA |
|  | **GP** | 91.9 | 98.4 | 100.0 | 100.0 | NA | 52.9 | 75.2 | 89.9 | 95.7 | 25.0 | 5.1 | 9.7 | NA | NA |
|  | **HL** | 91.3 | 97.8 | 100.0 | 100.0 | NA | 81.7 | 100.0 | 97.0 | 98.8 | 26.2 | 2.0 | 23.7 | 37.5 | NA |
|  | **Hca** | 91.1 | 98.1 | 100.0 | 100.0 | NA | 78.6 | 100.0 | 93.3 | 97.4 | 47.8 | 76.4 | 29.6 | 0.0 | NA |
|  | **Dca** | 90.3 | 97.4 | 100.0 | 100.0 | NA | 40.0 | 60.2 | 91.0 | 95.5 | 17.0 | 11.8 | 25.1 | 0.0 | NA |

**NA**: Not calculated due to the absence of compound on any of the plant tissues

**Table S13**. Calculated accumulation percentages of pharmaceuticals and their metabolites in plants after their simultaneous application.

| **Plant part** | **Soil type** | **Accumulation (%)** | | | | | | | | | | | | | |
| --- | --- | --- | --- | --- | --- | --- | --- | --- | --- | --- | --- | --- | --- | --- | --- |
|  |  | **CAR** | **EPC** | **TDC** | **OXC** | **DHC** | **CIT** | **DCIT** | **CLI** | **CLIS** | **FEX** | **IRB** | **SUL** | **N4AS** | **N1AS** |
| **Onion Roots** | **SChS** | 19.6 | 2.1 | 0.0 | 0.9 | NA | 86.0 | 89.7 | 37.4 | 36.9 | 89.8 | 95.1 | 57.7 | 100.0 | NA |
|  | **HCh** | 14.5 | 1.5 | 0.0 | 0.0 | NA | 81.2 | 91.5 | 36.4 | 32.7 | 91.7 | 91.9 | 63.4 | 100.0 | NA |
|  | **GP** | 13.1 | 1.9 | 0.0 | 0.6 | NA | 82.3 | 87.3 | 46.5 | 38.2 | 96.4 | 85.8 | 62.0 | 58.4 | NA |
|  | **HL** | 7.1 | 1.2 | 0.0 | 0.4 | NA | 77.4 | 79.0 | 41.5 | 37.8 | 94.3 | 93.3 | 65.0 | 100.0 | NA |
|  | **Hca** | 12.4 | 1.7 | 0.0 | 1.0 | NA | 86.8 | 86.6 | 46.5 | 52.3 | 91.3 | 98.4 | 64.1 | 100.0 | NA |
|  | **Dca** | 12.8 | 1.9 | 0.0 | 0.4 | NA | 87.7 | 92.8 | 51.5 | 61.0 | 96.6 | 99.2 | 77.8 | 100.0 | NA |
| **Onion Bulb** | **SChS** | 3.6 | 5.9 | 0.0 | 0.0 | NA | 0.5 | 0.0 | 2.8 | 1.2 | 0.5 | 4.3 | 19.6 | 0.0 | NA |
|  | **HCh** | 2.4 | 3.8 | 0.0 | 0.0 | NA | 0.6 | 0.0 | 3.0 | 0.9 | 0.4 | 7.1 | 16.5 | 0.0 | NA |
|  | **GP** | 4.0 | 6.3 | 0.0 | 4.4 | NA | 1.1 | 0.0 | 3.8 | 1.6 | 0.5 | 14.0 | 20.8 | 41.6 | NA |
|  | **HL** | 1.9 | 3.6 | 0.0 | 2.1 | NA | 0.6 | 0.0 | 1.9 | 0.7 | 0.4 | 6.4 | 13.3 | 0.0 | NA |
|  | **Hca** | 3.2 | 4.3 | 0.0 | 0.0 | NA | 0.4 | 0.0 | 2.4 | 0.8 | 0.3 | 0.3 | 11.2 | 0.0 | NA |
|  | **Dca** | 2.5 | 3.0 | 0.0 | 0.0 | NA | 0.6 | 0.0 | 2.8 | 1.0 | 0.2 | 0.3 | 9.9 | 0.0 | NA |
| **Onion Leaves** | **SChS** | 76.8 | 91.9 | 100.0 | 99.1 | NA | 13.4 | 10.3 | 59.8 | 61.9 | 9.7 | 0.6 | 22.7 | 0.0 | NA |
|  | **HCh** | 83.1 | 94.7 | 100.0 | 100.0 | NA | 18.2 | 8.5 | 60.6 | 66.5 | 7.8 | 1.0 | 20.0 | 0.0 | NA |
|  | **GP** | 82.9 | 91.8 | 100.0 | 95.0 | NA | 16.5 | 12.7 | 49.7 | 60.3 | 3.1 | 0.2 | 17.2 | 0.0 | NA |
|  | **HL** | 91.0 | 95.2 | 100.0 | 97.5 | NA | 22.1 | 21.0 | 56.6 | 61.5 | 5.4 | 0.3 | 21.7 | 0.0 | NA |
|  | **Hca** | 84.4 | 94.0 | 100.0 | 99.0 | NA | 12.8 | 13.4 | 51.1 | 46.9 | 8.4 | 1.3 | 24.7 | 0.0 | NA |
|  | **Dca** | 84.6 | 95.1 | 100.0 | 99.6 | NA | 11.8 | 7.2 | 45.7 | 37.9 | 3.2 | 0.4 | 12.3 | 0.0 | NA |
| **Spinach Roots** | **SChS** | - | - | - | - | - | - | - | - | - | - | - | - | - | - |
|  | **HCh** | 29.1 | 2.3 | 0.0 | NA | NA | 41.0 | 0.0 | 15.4 | 13.9 | 24.8 | 90.8 | 67.9 | 29.3 | NA |
|  | **GP** | 23.9 | 6.4 | 0.0 | NA | NA | 24.7 | NA | 18.0 | 12.6 | 20.5 | 97.6 | 64.6 | 53.0 | NA |
|  | **HL** | 28.0 | 3.7 | 0.0 | NA | NA | 72.9 | 27.4 | 30.0 | 11.9 | 29.2 | 95.6 | 41.8 | 15.9 | NA |
|  | **Hca** | 24.4 | 1.9 | 0.0 | NA | NA | 31.2 | 21.0 | 31.4 | 16.8 | 20.3 | 84.2 | 13.2 | 0.0 | NA |
|  | **Dca** | 30.5 | 2.3 | 0.0 | NA | NA | 89.3 | 100.0 | 65.0 | 33.8 | 65.0 | 95.2 | 51.6 | 45.7 | NA |
| **Spinach Leaves** | **SChS** | - | - | - | - | - | - | - | - | - | - | - | - | - | - |
|  | **HCh** | 70.9 | 97.7 | 100.0 | NA | NA | 59.0 | 100.0 | 84.6 | 86.1 | 75.2 | 9.2 | 32.1 | 70.7 | NA |
|  | **GP** | 76.1 | 93.6 | 100.0 | NA | NA | 75.3 | NA | 82.0 | 87.4 | 79.5 | 2.4 | 35.4 | 47.0 | NA |
|  | **HL** | 72.0 | 96.3 | 100.0 | NA | NA | 27.1 | 72.6 | 70.0 | 88.1 | 70.8 | 4.4 | 58.2 | 84.1 | NA |
|  | **Hca** | 75.6 | 98.1 | 100.0 | NA | NA | 68.8 | 79.0 | 68.6 | 83.2 | 79.7 | 15.8 | 86.8 | 100.0 | NA |
|  | **Dca** | 69.5 | 97.7 | 100.0 | NA | NA | 10.7 | 0.0 | 35.0 | 66.2 | 35.0 | 4.8 | 48.4 | 54.3 | NA |
| **Radish Roots + Bulbs** | **SChS** | 16.0 | 2.9 | 0.0 | 0.0 | NA | 66.8 | 23.6 | 13.5 | 8.7 | 48.3 | 80.8 | 67.8 | NA | NA |
|  | **HCh** | 11.8 | 3.2 | 0.0 | 0.0 | NA | 33.5 | 4.9 | 10.2 | 7.9 | 29.9 | 72.0 | 49.0 | 55.2 | NA |
|  | **GP** | 20.9 | 5.9 | 0.0 | 0.0 | NA | 74.3 | 49.8 | 19.4 | 9.5 | 87.4 | 98.5 | 85.9 | NA | NA |
|  | **HL** | 10.6 | 3.0 | 0.0 | 0.0 | NA | 73.9 | 31.1 | 16.3 | 8.1 | 73.0 | 83.8 | 53.9 | 39.8 | NA |
|  | **Hca** | 14.6 | 3.9 | 0.0 | 0.0 | NA | 24.0 | 0.0 | 10.8 | 2.2 | 26.7 | 38.6 | 42.9 | NA | NA |
|  | **Dca** | 11.5 | 2.6 | 0.0 | 0.0 | NA | 60.5 | 43.5 | 8.5 | 4.7 | 88.3 | 96.2 | 61.6 | 46.3 | NA |
| **Radish Leaves** | **SChS** | 84.0 | 97.1 | 100.0 | 100.0 | NA | 33.2 | 76.4 | 86.5 | 91.3 | 51.7 | 19.2 | 32.2 | NA | NA |
|  | **HCh** | 88.2 | 96.8 | 100.0 | 100.0 | NA | 66.5 | 95.1 | 89.8 | 92.1 | 70.1 | 28.0 | 51.0 | 44.8 | NA |
|  | **GP** | 79.1 | 94.1 | 100.0 | 100.0 | NA | 25.7 | 50.2 | 80.6 | 90.5 | 12.6 | 1.5 | 14.1 | NA | NA |
|  | **HL** | 89.4 | 97.0 | 100.0 | 100.0 | NA | 26.1 | 68.9 | 83.7 | 91.9 | 27.0 | 16.2 | 46.1 | 60.2 | NA |
|  | **Hca** | 85.4 | 96.1 | 100.0 | 100.0 | NA | 76.0 | 100.0 | 89.2 | 97.8 | 73.3 | 61.4 | 57.1 | NA | NA |
|  | **Dca** | 88.5 | 97.4 | 100.0 | 100.0 | NA | 39.5 | 56.5 | 91.5 | 95.3 | 11.7 | 3.8 | 38.4 | 53.7 | NA |

**NA**: Not calculated due to the absence of compound on any of the plant tissues

**Table S14**. Concentration of parent pharmaceuticals and selected metabolites detected in soil after their single application.

| **Plant** | **Soil type** | **Concentration (ng g^-1^)** | | | | | | | | | | | | | |
| --- | --- | --- | --- | --- | --- | --- | --- | --- | --- | --- | --- | --- | --- | --- | --- |
|  |  | **CAR** | **EPC** | **TDC** | **OXC** | **DHC** | **CIT** | **DCIT** | **CLI** | **CLIS** | **FEX** | **IRB** | **SUL** | **N4AS** | **N1AS** |
| Onion | **SChS** | 1300 | 110 | 2.9 | <LOQ | 20 | 180 | 1.3 | 200 | 130 | 220 | 800 | 96 | <LOQ | <LOQ |
|  | **HCh** | 890 | 32 | 1.2 | <LOQ | 13 | 420 | 3.7 | 200 | 81 | 350 | 290 | 40 | <LOQ | <LOQ |
|  | **GP** | 2100 | 110 | 2.9 | <LOQ | 72 | 4700 | 33 | 240 | 200 | 340 | 2000 | 49 | <LOQ | <LOQ |
|  | **HL** | 1900 | 150 | 3.7 | <LOQ | 38 | 380 | 4.3 | 630 | 350 | 370 | 550 | 65 | 0.74 | <LOQ |
|  | **HCa** | 730 | 38 | 1.6 | <LOQ | 12 | 1100 | 5.8 | 120 | 150 | 930 | 540 | 340 | 5.7 | <LOQ |
|  | **DCa** | 950 | 42 | 1 | <LOQ | 19 | 440 | 6.8 | 110 | 230 | 680 | 460 | 210 | 4 | <LOQ |
| Spinach | **SChS** | 340 | 8.4 | 0.81 | <LOQ | 10 | 61 | <LOQ | 110 | 74 | 530 | 430 | 39 | <LOQ | <LOQ |
|  | **HCh** | 540 | 9.9 | 1.2 | <LOQ | 14 | 660 | 2.8 | 240 | 92 | 320 | 350 | 36 | <LOQ | <LOQ |
|  | **GP** | 1600 | 100 | 5.7 | <LOQ | 15 | 160 | 1.1 | 110 | 73 | 240 | 550 | 40 | <LOQ | <LOQ |
|  | **HL** | 600 | 33 | 1.2 | <LOQ | 8.1 | 240 | 1.2 | 240 | 120 | 380 | 460 | 100 | 1.3 | <LOQ |
|  | **HCa** | 820 | 31 | 1.6 | <LOQ | 18 | 1500 | 7.9 | 420 | 410 | 400 | 630 | 150 | 2 | <LOQ |
|  | **DCa** | 2400 | 88 | 7.8 | <LOQ | 37 | <LOQ | <LOQ | 570 | 640 | 4300 | 770 | 210 | 1.9 | <LOQ |
| Radish | **SChS** | 940 | 52 | 1.2 | <LOQ | 24 | 340 | 2.7 | 210 | 120 | 130 | 880 | 37 | 0.76 | <LOQ |
|  | **HCh** | 960 | 23 | 1.6 | <LOQ | 22 | 250 | 1.5 | 1700 | 600 | 640 | 540 | 52 | 1 | <LOQ |
|  | **GP** | 760 | 38 | 0.82 | <LOQ | 13 | 1300 | 11 | 150 | 97 | 150 | 400 | 37 | 0.57 | <LOQ |
|  | **HL** | 820 | 48 | 1.2 | <LOQ | 29 | 84 | 0.82 | 170 | 60 | 430 | 510 | 56 | 1.4 | <LOQ |
|  | **HCa** | 710 | 22 | 0.82 | <LOQ | 7.4 | 150 | 0.95 | 240 | 240 | 840 | 650 | 120 | 2 | <LOQ |
|  | **DCa** | 570 | 32 | 3 | <LOQ | 5.9 | 2500 | 26 | 370 | 460 | 630 | 1300 | 130 | 2.6 | <LOQ |

**LOQ**: Limit of Quantification

**Table S15**. Concentration of parent pharmaceuticals and selected metabolites detected in soil after their simultaneous application.

| **Plant** | **Soil type** | **Concentration (ng g^-1^)** | | | | | | | | | | | | | |
| --- | --- | --- | --- | --- | --- | --- | --- | --- | --- | --- | --- | --- | --- | --- | --- |
|  |  | **CAR** | **EPC** | **TDC** | **OXC** | **DHC** | **CIT** | **DCIT** | **CLI** | **CLIS** | **FEX** | **IRB** | **SUL** | **N4AS** | **N1AS** |
| Onion | **SChS** | 910 | 82 | 2.2 | <LOQ | 11 | 340 | 4.5 | 200 | 110 | 180 | 770 | 55 | 0.52 | <LOQ |
|  | **HCh** | 880 | 16 | 0.27 | <LOQ | 11 | 620 | 4 | 360 | 88 | 560 | 610 | 62 | 0.43 | <LOQ |
|  | **GP** | 1200 | 61 | 1.4 | <LOQ | 13 | 490 | 3.8 | 240 | 150 | 320 | 730 | 50 | 0.45 | <LOQ |
|  | **HL** | 1400 | 100 | 2.1 | <LOQ | 18 | 330 | 3.2 | 580 | 300 | 850 | 940 | 160 | 2.7 | <LOQ |
|  | **HCa** | 820 | 26 | 1.2 | <LOQ | 7.7 | 410 | 2.2 | 200 | 180 | 240 | 410 | 160 | 1.2 | <LOQ |
|  | **DCa** | 1100 | 110 | 8.9 | <LOQ | 9.8 | 550 | 3.9 | 250 | 320 | 300 | 620 | 220 | 2.6 | <LOQ |
| Spinach | **SChS** | - | - | - | - | - | - | - | - | - | - | - | - | - | - |
|  | **HCh** | 410 | 4.8 | 0.27 | <LOQ | 3.4 | 98 | 0.38 | 170 | 32 | 170 | 340 | 55 | 0.31 | <LOQ |
|  | **GP** | 750 | 31 | 0.86 | <LOQ | 4.8 | 120 | 0.47 | 100 | 61 | 130 | 510 | 63 | 0 | <LOQ |
|  | **HL** | 620 | 29 | 0.52 | <LOQ | 4.5 | 90 | 0.56 | 120 | 89 | 240 | 400 | 73 | 1 | <LOQ |
|  | **HCa** | 890 | 18 | 0.86 | <LOQ | 6.9 | 240 | 0.92 | 300 | 140 | 290 | 500 | 250 | 2.2 | <LOQ |
|  | **DCa** | 1300 | 130 | 9.6 | <LOQ | 15 | 630 | 4 | 350 | 360 | 390 | 760 | 290 | 2.7 | <LOQ |
| Radish | **SChS** | 800 | 30 | 1.1 | <LOQ | 8.8 | 400 | 2.8 | 210 | 97 | 120 | 550 | 40 | 0.46 | <LOQ |
|  | **HCh** | 1100 | 12 | 0.27 | <LOQ | 15 | 740 | 3.7 | 550 | 96 | 740 | 730 | 75 | 0.59 | <LOQ |
|  | **GP** | 2000 | 40 | 0.85 | <LOQ | 43 | 2200 | 8.4 | 540 | 210 | 860 | 1500 | 140 | 2 | <LOQ |
|  | **HL** | 750 | 62 | 1.6 | <LOQ | 7.4 | 210 | 1.4 | 200 | 150 | 350 | 400 | 66 | 1.5 | <LOQ |
|  | **HCa** | 960 | 21 | 0.87 | <LOQ | 40 | 2500 | 10 | 780 | 420 | 660 | 720 | 210 | 3 | <LOQ |
|  | **DCa** | 1100 | 140 | 8.1 | <LOQ | 12 | 640 | 7.8 | 290 | 300 | 330 | 640 | 210 | 2.5 | <LOQ |

**LOQ**: Limit of Quantification; - Samples excluded due to cross-contamination while processing

**Table S16**. Bioaccumulation factor (BAF) of pharmaceuticals calculated after their single application.

| **Compound** | **Plant Tissues** | **SChS** | **HCh** | **GP** | **HL** | **HCa** | **DCa** |
| --- | --- | --- | --- | --- | --- | --- | --- |
| **CAR** | Onion Roots | 12.9 | 14.7 | 11.4 | 11.7 | 21.9 | 18.0 |
|  | Onion Bulbs | 0.9 | 2.0 | 1.2 | 1.9 | 2.9 | 2.3 |
|  | Onion Leaves | 20.6 | 61.3 | 30.0 | 62.6 | 81.8 | 67.7 |
|  | Spinach Roots | 0.4 | 1.7 | 2.0 | 9.0 | 2.0 | 0.8 |
|  | Spinach Leaves | 1.1 | 8.3 | 11.0 | 16.8 | 14.2 | 5.6 |
|  | Radish Roots + Bulbs | 3.2 | 3.0 | 9.7 | 8.8 | 4.7 | 6.4 |
|  | Radish Leaves | 27.6 | 20.1 | 107.1 | 71.3 | 43.9 | 56.1 |
| **CIT** | Onion Roots | 46.4 | 15.6 | 2.6 | 24.0 | 7.7 | 43.6 |
|  | Onion Bulbs | 0.1 | 0.1 | 0.05 | 0.1 | 0.02 | 0.1 |
|  | Onion Leaves | 3.4 | 2.8 | 0.1 | 3.4 | 0.5 | 2.5 |
|  | Spinach Roots | 1.1 | 0.1 | 3.2 | 2.2 | 0.1 | - |
|  | Spinach Leaves | 0.2 | 0.2 | 0.4 | 0.6 | 0.1 | - |
|  | Radish Roots + Bulbs | 1.1 | 2.4 | 0.5 | 1.6 | 0.6 | 0.4 |
|  | Radish Leaves | 1.4 | 2.2 | 0.5 | 5.6 | 1.6 | 0.2 |
| **CLI** | Onion Roots | 13.0 | 9.7 | 25.3 | 12.3 | 6.7 | 12.7 |
|  | Onion Bulbs | 0.2 | 0.1 | 0.2 | 0.1 | 0.1 | 0.1 |
|  | Onion Leaves | 3.1 | 3.2 | 7.4 | 4.5 | 3.1 | 3.4 |
|  | Spinach Roots | 0.1 | 1.1 | 0.2 | 0.3 | 0.3 | 0.3 |
|  | Spinach Leaves | 0.2 | 0.2 | 0.4 | 0.4 | 0.7 | 0.3 |
|  | Radish Roots + Bulbs | 0.1 | 0.3 | 0.1 | 0.7 | 0.2 | 0.2 |
|  | Radish Leaves | 1.2 | 2.5 | 0.7 | 6.1 | 5.6 | 1.7 |
| **FEX** | Onion Roots | 13.6 | 6.0 | 8.8 | 21.4 | 8.5 | 6.0 |
|  | Onion Bulbs | 0.01 | 0.03 | 0.02 | 0.1 | 0.01 | 0.01 |
|  | Onion Leaves | 0.1 | 0.05 | 0.1 | 0.4 | 0.2 | 0.1 |
|  | Spinach Roots | 0.1 | 0.3 | 0.3 | 0.6 | 0.4 | 0.1 |
|  | Spinach Leaves | 0.1 | 0.5 | 0.7 | 0.8 | 0.6 | 0.1 |
|  | Radish Roots + Bulbs | 0.3 | 0.2 | 0.3 | 0.2 | 0.1 | 0.1 |
|  | Radish Leaves | 0.1 | 0.1 | 0.1 | 0.05 | 0.04 | 0.02 |
| **IRB** | Onion Roots | 11.1 | 23.8 | 5.5 | 14.0 | 10.4 | 10.0 |
|  | Onion Bulbs | 0.1 | 0.3 | 0.1 | 0.2 | 0.01 | 0.01 |
|  | Onion Leaves | 0.04 | 0.1 | 0.004 | 0.01 | 0.01 | 0.02 |
|  | Spinach Roots | 0.4 | 1.5 | 0.9 | 2.0 | 0.3 | 0.1 |
|  | Spinach Leaves | 0.1 | 1.2 | 0.03 | 0.1 | 0.05 | 0.01 |
|  | Radish Roots + Bulbs | 0.8 | 1.7 | 2.5 | 0.8 | 0.3 | 0.5 |
|  | Radish Leaves | 0.04 | 0.02 | 0.1 | 0.01 | 0.8 | 0.1 |
| **SUL** | Onion Roots | 86.6 | 74.4 | 12.5 | 24.3 | 72.9 | 23.3 |
|  | Onion Bulbs | 5.9 | 5.0 | 0.6 | 1.6 | 4.7 | 0.7 |
|  | Onion Leaves | 5.9 | 6.6 | 0.9 | 1.5 | 7.0 | 1.1 |
|  | Spinach Roots | 9.8 | 8.1 | 0.3 | 3.0 | 7.8 | 0.3 |
|  | Spinach Leaves | 2.1 | 3.3 | 0.6 | 1.5 | 3.0 | 0.1 |
|  | Radish Roots + Bulbs | 8.8 | 6.6 | 1.9 | 5.8 | 5.7 | 2.6 |
|  | Radish Leaves | 0.9 | 1.6 | 0.5 | 3.5 | 2.6 | 0.8 |

**Table S17**. Bioaccumulation factor (BAF) of pharmaceuticals calculated after their simultaneous application.

| **Compound** | **Plant Tissues** | **SChS** | **HCh** | **GP** | **HL** | **HCa** | **DCa** |
| --- | --- | --- | --- | --- | --- | --- | --- |
| **CAR** | Onion Roots | 7.3 | 4.8 | 7.1 | 5.9 | 5.7 | 5.0 |
|  | Onion Bulbs | 1.0 | 0.7 | 1.0 | 0.6 | 0.5 | 0.4 |
|  | Onion Leaves | 33.4 | 35.7 | 34.7 | 16.4 | 23.0 | 28.0 |
|  | Spinach Roots | 2.3 | 2.2 | 0.8 | - | 0.9 | 0.5 |
|  | Spinach Leaves | 6.0 | 5.5 | 2.7 | - | 3.2 | 1.1 |
|  | Radish Roots + Bulbs | 1.8 | 3.8 | 0.8 | 2.6 | 1.5 | 1.8 |
|  | Radish Leaves | 4.6 | 27.2 | 4.0 | 13.9 | 9.3 | 18.8 |
| **CIT** | Onion Roots | 6.5 | 3.8 | 3.4 | 2.6 | 7.0 | 4.7 |
|  | Onion Bulbs | 0.01 | 0.01 | 0.01 | 0.01 | 0.01 | 0.01 |
|  | Onion Leaves | 0.3 | 0.3 | 0.3 | 0.3 | 0.5 | 0.3 |
|  | Spinach Roots | 0.00 | 0.1 | 0.01 | 0.2 | 0.03 | 0.2 |
|  | Spinach Leaves | 0.00 | 0.04 | 0.02 | 0.05 | 0.03 | 0.01 |
|  | Radish Roots + Bulbs | 0.5 | 0.2 | 0.2 | 0.3 | 0.02 | 0.4 |
|  | Radish Leaves | 0.2 | 0.3 | 0.0 | 0.1 | 0.1 | 0.2 |
| **CLI** | Onion Roots | 1.5 | 1.3 | 2.8 | 1.5 | 1.2 | 1.9 |
|  | Onion Bulbs | 0.03 | 0.02 | 0.03 | 0.02 | 0.02 | 0.02 |
|  | Onion Leaves | 0.9 | 0.8 | 1.2 | 0.8 | 0.9 | 0.7 |
|  | Spinach Roots | 0.0 | 0.1 | 0.1 | 0.00 | 0.04 | 0.05 |
|  | Spinach Leaves | 0.1 | 0.2 | 0.1 | 0.00 | 0.1 | 0.03 |
|  | Radish Roots + Bulbs | 0.02 | 0.1 | 0.01 | 0.1 | 0.1 | 0.04 |
|  | Radish Leaves | 0.1 | 0.4 | 0.1 | 0.5 | 0.4 | 0.4 |
| **FEX** | Onion Roots | 1.8 | 2.0 | 1.8 | 1.8 | 2.7 | 1.7 |
|  | Onion Bulbs | 0.002 | 0.002 | 0.002 | 0.002 | 0.003 | 0.001 |
|  | Onion Leaves | 0.1 | 0.1 | 0.03 | 0.04 | 0.1 | 0.03 |
|  | Spinach Roots | 0.00 | 0.1 | 0.02 | 0.2 | 0.1 | 0.1 |
|  | Spinach Leaves | 0.00 | 0.1 | 0.04 | 0.2 | 0.1 | 0.01 |
|  | Radish Roots + Bulbs | 0.1 | 0.04 | 0.03 | 0.1 | 0.01 | 0.1 |
|  | Radish Leaves | 0.1 | 0.1 | 0.003 | 0.03 | 0.03 | 0.01 |
| **IRB** | Onion Roots | 3.5 | 3.7 | 3.0 | 1.8 | 3.0 | 1.8 |
|  | Onion Bulbs | 0.04 | 0.1 | 0.1 | 0.04 | 0.00 | 0.00 |
|  | Onion Leaves | 0.01 | 0.02 | 0.003 | 0.002 | 0.02 | 0.004 |
|  | Spinach Roots | 0.00 | 0.4 | 0.6 | 0.8 | 0.1 | 0.1 |
|  | Spinach Leaves | 0.00 | 0.02 | 0.01 | 0.02 | 0.01 | 0.002 |
|  | Radish Roots + Bulbs | 0.2 | 0.6 | 0.1 | 0.2 | 0.02 | 0.2 |
|  | Radish Leaves | 0.1 | 0.2 | 0.001 | 0.03 | 0.03 | 0.004 |
| **SUL** | Onion Roots | 0.9 | 1.1 | 1.3 | 1.4 | 2.5 | 2.2 |
|  | Onion Bulbs | 0.1 | 0.1 | 0.1 | 0.1 | 0.1 | 0.1 |
|  | Onion Leaves | 0.1 | 0.1 | 0.2 | 0.2 | 0.4 | 0.2 |
|  | Spinach Roots | 0.00 | 0.7 | 0.3 | 0.3 | 0.05 | 0.1 |
|  | Spinach Leaves | 0.00 | 0.2 | 0.1 | 0.2 | 0.1 | 0.02 |
|  | Radish Roots + Bulbs | 0.1 | 0.1 | 0.04 | 0.1 | 0.02 | 0.1 |
|  | Radish Leaves | 0.04 | 0.1 | 0.004 | 0.1 | 0.02 | 0.1 |
